# The National Health Service urgent cancer referral pathway for suspected urological cancers: early economic evaluation of a risk prediction test

**DOI:** 10.1017/S0266462324000023

**Published:** 2024-01-12

**Authors:** Paola Cocco, Alison Florence Smith, Richard D. Neal, Bethany Shinkins

**Affiliations:** 1 Academic Unit of Health Economics, Leeds Institute of Health Sciences (LIHS), University of Leeds, Leeds, UK; 2 Department of Health and Community Sciences, Faculty of Health and Life Sciences, University of Exeter, Exeter, UK; 3 Division of Health Sciences, University of Warwick, Coventry, UK

**Keywords:** early economic evaluation, urological cancer, bladder cancer, renal cancer, prostate cancer, 2-week wait, urgent referral

## Abstract

**Objectives:**

In the UK, the number of patients urgently referred for suspected cancer is increasing, and providers are struggling to cope with demand. We explore the potential cost-effectiveness of a new risk prediction test – the PinPoint test – to triage and prioritize patients urgently referred with suspected urological cancers.

**Methods:**

Two simulation models were developed to reflect the diagnostic pathways for patients with (i) suspected prostate cancer, and (ii) bladder or kidney cancer, comparing the PinPoint test to current practice. An early economic analysis was conducted from a UK National Health Service (NHS) perspective. The primary outcomes were the percentage of individuals seen within 2 weeks and health care costs. An exploratory analysis was conducted to understand the potential impact of the Pinpoint test on quality-adjusted life years gained.

**Results:**

Across both models and applications, the PinPoint test led to more individuals with urological cancer being seen within 2 weeks. Using PinPoint only to prioritize patients led to increased costs overall, whereas using PinPoint to both triage and prioritize patients led to cost savings. The estimated impact on life years gained/lost was very small and highly uncertain.

**Conclusions:**

Using the PinPoint test to prioritize urgent referrals meant that more individuals with urological cancer were seen within 2 weeks, but at additional cost to the NHS. If used as a triage and prioritization tool, the PinPoint test shortens wait times for referred individuals and is cost saving. More data on the impact of short-term delays to diagnosis on health-related quality of life is needed.

## Introduction

In the UK, the National Institute for Health Care Excellence (NICE) recommends the patient characteristics and symptoms that warrant an urgent cancer referral from primary care to secondary care for further investigation within 2 weeks (1).

Over the past 10 years, the number of urgent referrals for suspected urological cancer has increased – this might be due to several awareness campaigns for urological cancers or a lower threshold for referral (2). This has taken a toll on the ability of providers to meet the 2-week wait (TWW) national target set by NHS England (93 percent of patients to be seen within 2 weeks) (3). For the first three quarters of 2022–2023, the TWW target was not been met for the suspected urological cancer pathway on average across all English providers (4). There is variation between providers, both in terms of volume of referrals and their ability to meet the TWW operational target – perhaps due to different clinical schedules in secondary care, the use of locum doctors, workforce gaps, or differences in clinical decision-making.

Given that most referred individuals do not receive a cancer diagnosis, there is scope for better triage and prioritization for those at greatest risk of cancer. PinPoint Data Science Ltd. have developed a multivariable machine learning algorithm, hereafter referred to as the PinPoint test, to predict the risk of cancer in symptomatic patients referred urgently from primary care (5). The test has been designed to determine patients’ risk of cancer based on several routine blood tests (i.e., hematological, biochemical, and tumor markers). There are two main applications for PinPoint test: (i) identifying and prioritizing individuals at high risk of having cancer for urgent referral (i.e., “prioritization” use case); and (ii) safely ruling out individuals with a very low risk of having cancer while prioritizing individuals with a high risk of having cancer for urgent referral (i.e. “triage and prioritization” use case). The diagnostic accuracy of the test has been validated in a retrospective diagnostic prediction study (5). The test is currently being prospectively validated across multiple Cancer Alliances in England.

The aim of this study was to explore, via early economic modeling, the potential cost-effectiveness of using the PinPoint test as a means to (i) prioritize; and (ii) triage and prioritize individuals meeting the symptom criteria for urgent referral for suspected urological cancer compared to standard care.

## Methods

Two resource-constrained discrete event simulation models were developed in SIMUL8 to track adult individuals presenting to general practice with symptoms prompting urgent referral for urological cancer (excluding testicular cancer), including (i) prostate cancer; (ii) bladder or kidney cancer, to diagnosis in secondary care. All patients exit the models with or without a diagnosis of urological cancer.

A technical description of the models is provided in Supplementary materials S1 and S2.

## Model structure

**Figure 1.**
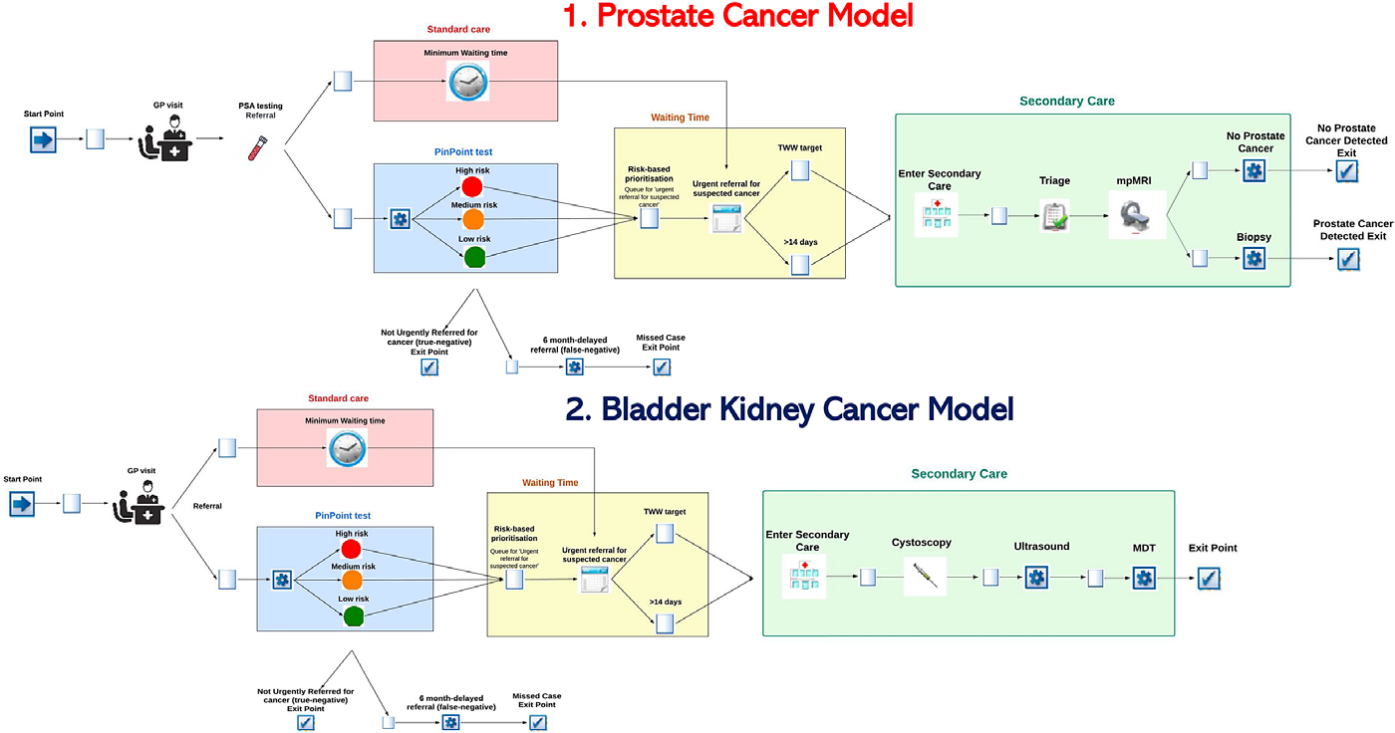
Simplified schematic of the structure of the prostate cancer model, and the bladder/kidney cancer model, separately.

Figure 1 presents a schematic of the structures of the prostate cancer model and bladder/kidney cancer model. Both models compare standard care to a pathway that uses the PinPoint test as a triage and/or prioritization tool. The focus of both models is the urgent suspected cancer pathways for urological cancers (i.e., referrals to secondary care for further diagnostic investigation within 2 weeks).

While there are some key similarities across both models (e.g., waiting time queue for patients not meeting the TWW target), the pathways in primary and secondary care differ in terms of the diagnostic tests run, and it was therefore decided to develop two separate models.

Consultations with a clinical expert [RN, General Practitioner (GP)] and multiple urology clinicians, as well as a review of NICE clinical guidelines (1;6;7), informed the model structure and parameterization. As the PinPoint test is yet to be evaluated as an intervention (i.e., used to change patient management decisions), we also sought clinical opinion on the expected health impact of the test.

### Standard care (prostate cancer)

All individuals entering the model have a prostate-specific antigen (PSA) test ordered. They are then assumed to have a PSA above the age-specific threshold and are therefore urgently referred to secondary care for further diagnostic investigation (1). In secondary care, patients first undergo triage and are then offered multi-parametric magnetic resonance imaging (mpMRI) to identify individuals who need to undergo a transrectal ultrasound-guided (TRUS) biopsy. All patients with positive biopsy results are reviewed in a multidisciplinary team (MDT) meeting to confirm cancer diagnosis.

### Standard care (bladder and kidney cancer)

All adult individuals entering the model are assumed to have symptoms suggestive of bladder or kidney cancer (i.e., visible haematuria) and therefore are urgently referred to secondary care for further investigation (1). In secondary care, patients are tested with cystoscopy, ultrasound, and X-ray to diagnose bladder or kidney cancer. The results of the tests are reviewed in an MDT meeting to determine diagnosis.

### PinPoint test

Across both models, the PinPoint test is ordered by the GP for all those who meet the NICE suspected urological cancer referral criteria (1). Each patient is assigned an individual risk of having cancer, upon which individuals can be categorized into three groups: high, medium, and low risk. In the prioritization use case, all individuals are urgently referred, and those who are categorized as high risk by the PinPoint test are seen first, followed by those who are categorized as medium risk, then low risk. In this context, the key role of the PinPoint test is to reduce waiting times for those with the highest likelihood of having cancer.

Alternatively, in the triage and prioritization use case, individuals who are categorized as low risk are not referred down the urgent cancer pathway and only individuals classed as high and medium risk are urgently referred to secondary care – with high-risk individuals being seen first, followed by medium-risk individuals. The key benefit of PinPoint as a triage and prioritization tool is to expedite the waiting time for individuals with higher risk of having cancer while also reducing the overall volume of urgent referrals.

We sought clinical opinion on what might happen to individuals who receive a true-negative PinPoint test (i.e., individuals without cancer classified as low risk). For the prostate pathway, many men will have benign prostatic hyperplasia. Most of these can be managed solely in primary care, but some may need non-urgent referral to urology for further assessment. Others may have chronic prostatitis, recurrent urinary infections, or bladder infection/inflammation and some may need nonurgent referral. For the bladder and renal pathway, likely non-cancer diagnoses are infection/inflammation, ureteric stones, nephritis, and nephropathies. While many of these patients will be managed in primary care, some may need referral to secondary care for assessment. Given the paucity of data to evidence what would happen to these individuals and the intended use of PinPoint test, no costs, benefits, or harms associated with receiving a true-negative result with PinPoint test have been included. There is clearly a need for further research on this to validate this assumption.

Although the threshold for “low risk” has been optimized to minimize the proportion of individuals with cancer misclassified as “low risk,” there will be a small proportion of individuals with cancer who are not urgently referred for suspected urological cancer. Cancer patients incorrectly not referred down the urgent cancer pathway are assumed to remain symptomatic, visit the GP twice for additional consultations, and receive a delayed referral to secondary care after 6 months.

Under both use cases for PinPoint test, depending on their cancer risk profile, patients are urgently referred and/or prioritized to be fast-tracked for diagnostic investigations in secondary care.

See Supplementary material S1 for a schematic of the implementation pathway for PinPoint test.

## Model parameters

Table 1 presents the parameters applied to the models (see Supplementary material S1 for full details).

**Table 1.** Parameters and related sources common to both models, and parameters specific to the prostate cancer model and bladder/kidney cancer model, separately

Parameter	Deterministic estimate	Data source
*Parameters common to the prostate and kidney/renal bladder cancer models*		
Urological cancer prevalence	16.9%	(8;14)
Diagnostic sensitivity PinPoint test (prioritization) – urological pathway	90.04%	(5)
Diagnostic specificity PinPoint test (prioritization) – urological pathway	35.48%	(5)
Diagnostic sensitivity PinPoint test (triage) – urological pathway	96.81%	(5)
Diagnostic specificity PinPoint test (triage) – urological pathway	20.02%	(5)
Cost of GP consultation	£45.44^a^	
Cost of multidisciplinary cancer team meeting	£165.82^a^	
*Parameters specific to the prostate cancer model*		
Proportion of prostate cancer referrals among urological referrals	75.91%	(8;14), expert opinion
Age-stratified proportions of patients with prostate cancer referred using TWW pathway	30–39 years old = 0.01% 40–49 years old = 0.94% 50–59 years old = 9.63% 60–69 years old = 32.95% 70–79 years old = 38.22% ≥80 years old = 18.24%	(8;14)
Diagnostic sensitivity for mpMRI	100%	Assumption
Diagnostic specificity for mpMRI	100%	Assumption
Diagnostic sensitivity for TRUS biopsy	100%	Assumption
Diagnostic specificity for TRUS biopsy	100%	Assumption
Cost of PSA testing	£5.68^a^	
Cost of PinPoint test	£35.17^a^	Internal communication with the company – Saving on phlebotomy costs
Cost of triage	£29.13^a^	
Cost of mpMRI	£341.86^a^	
Cost of TRUS biopsy	£927.91^a^	
*Parameters specific to the bladder/kidney cancer model*		
Proportion of bladder/kidney cancer referrals among urological referrals	24.09%	Expert opinion
Proportion of bladder cancer referrals among bladder/kidney referrals	59.79%	(8;14)
Proportion of kidney cancer referrals among bladder/kidney referrals	40.21%	(8;14)
Age-stratified proportions of patients with bladder cancer referred using TWW pathway	30–39 years old = 0.32% 40–49 years old = 2.10% 50–59 years old = 7.36% 60–69 years old = 23.40% 70–79 years old = 36.24% ≥80 years old = 30.58%	(8;14)
Age-stratified proportions of patients with kidney cancer referred using TWW pathway	30–39 = 2.29% 40–49 = 8.14% 50–59 = 17.67% 60–69 = 27.75% 70–79 = 27.47% ≥80 = 16.68%	(8;14)
Diagnostic sensitivity for cystoscopy	100%	Assumption
Diagnostic specificity for cystoscopy	100%	Assumption
Diagnostic sensitivity for Xray	100%	Assumption
Diagnostic specificity for Xray	100%	Assumption
Diagnostic sensitivity for ultrasound	100%	Assumption
Diagnostic specificity for ultrasound	100%	Assumption
Cost of PinPoint test	£39.17^a^	Internal communication with the company – Including phlebotomy costs
Cost of cystoscopy	£306.67^a^	
Cost of X-ray	£51.48^a^	
Cost of ultrasound	£78.35^a^	

a We used the Bank of England inflator to better reflect the notable increases seen in the inflation rates during the time of the analysis (autumn 2022 to winter 2023).

### Cancer prevalence

Route to diagnosis data (2013–2016) were used to inform the prevalence of urological cancer, and the proportion of urological referrals specific to cancer subgroup (8). The prevalence of urological cancer was set to 16.9 percent based on the reported number of confirmed urological cancer patients. The proportion of urgent urological referrals specific to prostate cancer was set to 75.91 percent, whereas the remaining portion was for bladder and kidney cancer. Of these, 59.79 percent of urgent referrals were specific for bladder cancer whereas the remaining portion was for kidney cancer.

### Number of urgent cancer referrals, capacity, and waiting times

The number of individuals urgently referred to the suspected urological cancer pathway each month and the ability of the providers to see each individual within 2 weeks is based on NHS Waiting Time Statistics (December 2021 to November 2022; (9)). While the numbers of patients urgently referred for (i) prostate cancer and (ii) bladder or kidney cancer feeding into the models differed depending on the subgroups of patients being urgently referred for each specific cancer, the same proportions of patients meeting the TWW in the suspected urological cancer pathway were applied.

Providers were categorized both by the volume of referrals and by the proportion of patients seen within 2 weeks (hereafter referred to as “performance”). For each scenario, we calculated the number of individuals urgently referred for suspected urological cancer, the proportion of individuals meeting the TWW target, and the distribution of additional waiting days for those who could not be seen within 2 weeks (9).

This data was used to underpin the waiting times and capacity inputs in the model. Where demand exceeds available capacity, some individuals experience an additional delay (beyond 14 days) ranging between 1 and 16 days depending on the distribution of additional waiting days for those patients not seen within 2 weeks.

### Costs

The analysis adopted a UK NHS cost perspective. The cost (£) of each test in the diagnostic pathway, the GP consultations, and secondary care outpatient appointments were derived from NHS Reference Costs 2019/2020 (10), the NHS National Tariffs 2020/2021 (11), and the Personal Social Services Research Unit (PSSRU) unit costs (12). The cost of the PinPoint test was based on internal communication with the manufacturer. Costs were inflated to 2022 using the Bank of England inflator where appropriate (13).

## Model analysis

The models cover a 1-year period between December 2021 and November 2022, a different estimate for patients urgently referred, the maximum number of patients seen within 2 weeks, and the distribution of additional waiting days for those who were not seen within 2 weeks. Given the short time horizon, no discount rate was applied.

Primary outcomes of the model analysis were (i) the percentage of patients seen within 2 weeks, and (ii) total healthcare costs associated with the diagnosis. We investigated the relative cost-effectiveness of PinPoint test compared to usual care while capturing the impact of both (i) the volume of referred patients and (ii) the providers’ “performance.”

All deterministic analyses are based on running 650 model replications (i.e., running the model 650 times using the same deterministic estimates for model parameters, with each run using a different random number sequence) to minimize the impact of first-order uncertainty on the model results.

For each scenario, a probabilistic sensitivity analysis (PSA) was also conducted running 1,200 replications. The number of deterministic and probabilistic trials was set to values sufficient to provide stable model outputs (see Supplementary material S1).

## Exploratory analysis

We also explored how longer delays to diagnosis may impact the health outcomes of those individuals with cancer. We applied estimates from a UK-based modeling study that assessed the impact of 2-, 4- and 6-month delays in referral on 10-year survival for different cancer subgroups (stratified by age) (14). To apply these estimates, we used the age groups for each cancer pathway as reported in the Routes to Diagnosis data 2013–2016 (8) (Table 1). Life years lost were discounted at 3.5 percent per year based on the NICE discount rate.

In the models, individuals with cancer who have to wait longer than 28 days are assumed to experience a 2-month delay decrement to their 10-year survival due to the detrimental impact of waiting for cancer treatment. Similarly, in the triage and prioritization use case, missed cases are assumed to experience a 6-month delay decrement to their 10-year survival following an incorrect low-risk result. The total life years gained (LYG) were estimated by comparing total years of life lost between standard care and PinPoint test arm.

In addition, to enable the standard cost-effectiveness calculations to be undertaken, we estimated the incremental net monetary benefit (INMB) by comparing the PinPoint test to standard care, using the NICE willingness-to-pay (WTP) threshold of £20,000 per quality-adjusted life year (QALY) gained. There is currently, however, a lack of data on the impact of short-term delays to diagnosis of cancer on long-term health-related quality-of-life (HRQoL). We therefore assumed that a delayed or faster diagnosis in patients with cancer does not affect the quality of life – the QALYs gained are therefore based solely on the LYG.

## Results

This section describes the deterministic results for both models focusing on two scenarios: (i) poorly efficient providers with high volume of referrals and (ii) highly efficient providers with low volume of referrals. Additional results are reported in Supplementary material S3.

## TWW target

Across both models, PinPoint test as a prioritization tool does not alter the ability of providers to meet the TWW target for all urgently referred individuals (regardless of cancer status) (see Table 2), as the testing strategy does not release additional capacity since all individuals are still eventually referred with urgency. It does however ensure that those with cancer are seen within 2 weeks compared to standard care across all scenarios. In scenario 2, the TWW target of 93 percent is already being met in standard care, and the PinPoint test offers marginal benefit (e.g. the percentage seen within 2 weeks increases from 99 percent to 99.7 percent for those with cancer). The benefit is much larger in the context of poorly performing providers (high volume = 70 percent to 88 percent for patients with cancer). By prioritizing those with cancer, the proportion of non-cancers seen within 2 weeks is consequently lower.

**Table 2.** Results for prostate cancer model and bladder and kidney cancer model in the context of poorly efficient providers with high volumes of referrals, and highly efficient providers with low volume of referrals: average number of patients entering the model, average estimates for referral patterns (%) for standard care and PinPoint test (both use cases) across 650 deterministic model replications, sorted by volume of referrals and performance scenarios

Prostate cancer model							
		High volume sites			Low volume sites		
		Poorly efficient			Highly efficient		
		Standard care	PP – prioritization	PP – triage and prioritization	Standard care	PP – prioritization	PP – triage and prioritization
Patient cohort	All	2546			782		
	Cancer	429 (16.8%)			132 (16.8%)		
	No cancer	2118 (83.8%)			651 (84%)		
Referral	All	2546 (100%)	2546 (100%)	2109 (82.8%)	782 (100%)	782 (100%)	647 (82.8%)
	Cancer	428.5 (16.83%)	428.5 (16.83%)	416 (19.7%)	132 (16.8%)	132 (16.8%)	128 (19.7%)
	No cancer	2117.6 (83.18%)	2117.6 (83.18%)	1693 (80.3%)	651 (83.2%)	651 (83.2%)	520 (80.3%)
TWW achieved	All	1783 (70%)	1783 (70%)	1783 (84.6%)	771 (99%)	771 (99%)	647.4 (100%)
	Cancer	300.4 (70%)	382.4 (88%)	383 (92.1%)	129.8 (99%)	131.3 (99.7%)	127.5 (100%)
	No cancer	1482.7 (70%)	1400.7 (66.1%)	1400 (82.7%)	641.3 (99%)	639.8 (99%)	520 (100%)
Delayed referrals (seen after 2 weeks)	All	763 (30%)	763 (30%)	326 (15.5%)	11 (1%)	11 (1%)	0 (0%)
	Cancer	128.2 (30%)	46.1 (11%)	33 (7.9%)	1.9 (1%)	0.4 (0.2%)	0 (0%)
	No cancer	634.9 (30%)	717 (33.9%)	293 (17%)	9.2 (1%)	10.7 (1%)	0 (0%)
Significantly delayed referrals (seen after 28 days)	All	67.5 (8.8%)	67.5 (8.8%)	30 (9.1%)	0.5 (4.16%)	0.5 (4.1%)	0 (0%)
	Cancer	11.322 (8.8%)	4.3 (9%)	3 (9.2%)	0.077 (4.21%)	0.02 (6%)	0 (0%)
	No cancer	56 (8.8%)	63 (8.8%)	27 (9.1%)	0.38 (4.15%)	0.437 (4%)	0 (0%)
False negative delayed referrals (6 months)		0 (0%)	0 (0%)	14 (0.6%)	0 (0%)	0 (0%)	4 (0.5%)
*Bladder and kidney cancer model*							
Patient cohort	All	808			247		
	Cancer	136 (16.8%)			42 (16.8%)		
	No cancer	672 (84%)			206 (81.4%)		
Referral	All	808 (100%)	808 (100%)	669 (82.8%)	247 (100%)	247 (100%)	204 (82.7%)
	Cancer	136 (100%)	136 (100%)	133 (19.8%)	42 (100%)	42 (100%)	41 (19.8%)
	No cancer	672 (100%)	672 (100%)	536 (79.9%)	206 (100%)	206 (100%)	163 (79.7%)
TWW achieved	All	565 (70%)	565 (70%)	565 (84.5%)	246 (99.6%)	246 (99.6%)	204 (99.9%)
	Cancer	95 (70%)	121 (88%)	122 (91.6%)	41.3 (99%)	41.4 (99.9%)	41 (100%)
	No cancer	470 (70%)	445 (66.1%)	443 (82.7%)	204.8 (99.6%)	204.6 (99.5%)	163 (99.9%)
Delayed referrals (seen after 2 weeks)	All	243 (30%)	243 (30.1%)	104 (15.6%)	1 (0.4%)	1 (0.4%)	0.1 (0.02%)
	Cancer	41 (30%)	15 (11.4%)	11 (8.4%)	0.2 (0.5%)	0.1 (0.1%)	0.1 (0.01%)
	No cancer	202 (30%)	228 (33.9%)	93 (17.3%)	0.9 (0.5%)	1 (0.5%)	0.1 (0.02%)
Significantly delayed referrals (seen after 28 days)	All	19.8 (8.1%)	19.8 (8.1%)	10 (9.3%)	0.1 (4.9%)	0.1 (4.9%)	0.1 (13.35%)
	Cancer	3.4 (8.2%)	1.4 (9%)	1 (9.3%)	0.1 (5.4%)	0.1 (6.9%)	0 (0%)
	No cancer	16.5 (8.1%)	18.5 (8.1%)	9 (9.2%)	0.1 (4.8%)	0.1 (4.8%)	0.1 (15.4%)
False Negative delayed referrals (6 months)		0 (0%)	0 (0%)	4 (0.6%)	0 (0%)	0 (0%)	1.3 (0.4%)

*Note*: Although the numbers referred for (i) prostate cancer and (ii) bladder and kidney cancer differ, the estimated percentages for the proportion of individuals with suspected urological cancer seen within 2 weeks are virtually the same across both models.

Using the PinPoint test as a triage and prioritization tool increases the proportion of patients seen within 2 weeks (regardless of the cancer status), since individuals classed as low risk are not urgently referred thereby leading to additional capacity. Implementing the PinPoint test as a triage and prioritization tool provides the greatest benefit in the context of poorly efficient providers – in scenario 1, the percentage of patients seen within 2 weeks increases from 70 percent to 84.6 percent. The incremental benefit compared to standard care is reduced in scenario 2 (from 99 percent to 100 percent of patients seen within 2 weeks). This increased capacity in meeting the TWW target, however, is at the expense of a small proportion of individuals with cancer who are classed as low risk (approximately 3 percent of those with cancer).

## Healthcare costs

Across both models, using the PinPoint test for prioritization led to higher costs compared to standard care across all scenarios (see Tables 3 and 4). For prostate cancer, the PinPoint test resulted in an incremental mean annual cost ranging between £27,503 and £89,543 depending on the scenario being evaluated, and an incremental mean cost per patient of approximately £35.20 across all scenarios. In the bladder/kidney cancer model, the PinPoint test led to an incremental mean annual cost ranging between £9,675 and £31,650 depending on the scenario. Across both models and all scenarios, the incremental mean cost per patient reflected the additional costs of running the PinPoint test in primary care.

**Table 3.** Mean costs (95% CI), life years lost (95% CI), incremental costs and incremental life years gained (95% CI), incremental net monetary benefit (95% CI), mean cost per life years gained per total cohort and per patient for each testing option being evaluated in the prostate cancer model across 650 deterministic model replications, in the context of (i) poorly efficient providers with high volume of referrals; and (ii) highly efficient providers with low volume of referrals

		Standard care	PP test – prioritization	PP test – triage and prioritization
*Scenario 1: Poorly efficient sites with high volumes of referrals*				
Mean costs (95% CI)	Total cohort	£1,625,117 (£1,623,350; £1,626,884)	£1,713,473 (£1,711,560; £1,715,385)	£1,558,780 (£1,556,842; £1,560,718)
	Per patient	£638	£673	£612
Mean life years lost (95% CI)	Total cohort	0.034551 (0.0322; 0.0369)	0.12 (0.01096; 0.01391)	0.03636 (0.03421; 0.03852)
	Per patient	0.000081	0.000029	0.000085
Incremental costs (95% CI)	Total cohort		£89,543 (£89,543; £89,543)	−£66,336 (−£66,881; −£65,792)
	Per patient		£35.20	−£26.00
Incremental life years gained (95% CI)	Total cohort		0.0221 (0.0193431; 0.0249)	−0.000022 (−0.000065; −0.000021)
	Per patient		0.000052	−0.000004
INMB £20,000 WTP per QALY gained (95% CI)	Total cohort		−£89,100 (−£89,156; −£89,045)	£66,335 (£65,791; £66,660)
*Scenario 2: Highly efficient sites with low volumes of referrals*				
Mean costs (95% CI)	Total cohort	£498,784 (£497,767; £499,801)	£526,287 (£525,269; £527,304)	£478,193 (£477,058; £479,328)
	Per patient	£638	£673	£612
Mean life years lost (95% CI)	Total cohort	0.000305 (0.00080; 0.00053)	0.000087 (−0.000034; 0.0000208)	0.00821 (0.007161; 0.00926)
	Per patient	0.0000023	0.0000007	0.0000622
Incremental costs (95% CI)	Total cohort		£27,503 (£27,503; £27,503)	−£20,590 (−£22,130; −£19,050)
	Per patient		£35.20	−£26.33
Incremental life years gained (95% CI)	Total cohort		0.0000218 (−0.000038; 0.000474)	−0.0079 (−0.00899; −0.00683)
	Per patient		0.0000017	−0.0000599
INMB £20,000 WTP per QALY gained (95% CI)	Total cohort		−£27,499 (−£27,503; −£27,493)	£20,432 (£18,891; £21,973)

**Table 4.** Mean costs (95% CI), life years lost (95% CI), incremental costs and incremental life years gained (95% CI), incremental net monetary benefit (95% CI), mean cost per life years gained per total cohort and per patient for each testing option being evaluated in the bladder and kidney cancer model across 650 deterministic model replications, in the context of (i) poorly efficient providers with high volume of referrals; and (ii) highly efficient providers with low volume of referrals

		Standard care	PP test - prioritization	PP test - triage and prioritization
*Scenario 1: Poorly efficient sites with high volumes of referrals*				
Mean costs (95% CI)	Total cohort	£675,440 (£675,440; £675,440)	£707,089 (£707,089; £707,089)	£600,671 (£600,007; £601,335)
	Per patient	£836	£876	£743
Mean life years lost (95% CI)	Total cohort	0.326889 (0.308491; 0.345307)	0.126188 (0.114809; 0.137566)	1.535754 (1.470938; 1.60057)
	Per patient	0.002404	0.000928	0.011292
Incremental costs (95% CI)	Total cohort		£31,650 (£31,650; £31,650)	−£74,786 (−£75,450; −£74,122)
	Per patient		£39.17	−£92.54
Incremental life years gained (95% CI)	Total cohort		0.200711 (0.180758; 0.220665)	−1.20871 (−1.14192; −1.2755)
	Per patient		0.001476	−0.008889
INMB £20,000 WTP per QALY gained (95% CI)	Total cohort		−£27,635 (−£28,034; −£27,236)	£50,612 (£49,099; £52,123)
*Scenario 2: Highly efficient sites with low volumes of referrals*				
Mean costs (95% CI)	Total cohort	£206,477 (£206,477; £206,477)	£216,153 (£216,153; £216,153)	£183,437 (£183,071; £183,803)
	Per patient	£836	£876	£743
Mean life years lost (95% CI)	Total cohort	0.0008 (0.00154; 0.00005)	0.00025 (−0.00022; 0.00072)	0.402926 (0.36085; 0.436766)
	Per patient	0.0000267	0.0000060	0.0135233
Incremental costs (95% CI)	Total cohort		£9,675 (£9,675; £9,675)	−£23,033 (−£23,399; −£22,667)
	Per patient		£39.17	−£93.28
Incremental life years gained (95% CI)	Total cohort		0.000545 (−0.0003367; 0.00143)	−0.40213 (−0.43599; −0.36827)
	Per patient		0.0000207	−0.0134967
INMB £20,000 WTP per QALY gained (95% CI)	Total cohort		−£9,664 (−£9,682; −£9,646)	£14,991 (£14,202; £115,779)

PinPoint test used as a triage and prioritization tool, on the contrary, was a cost-saving strategy compared to standard care across both models (see Tables 3 and 4). In the prostate cancer model, the PinPoint test resulted in an incremental mean annual cost ranging between −£20,590 and −£66,336, and an incremental mean cost saving per patient ranging between £26 depending on the scenario. In the bladder/ kidney cancer model, the PinPoint test led to mean annual cost-savings ranging between −£23,040 and −£74,786, depending on the scenario.

## Exploratory analysis: life years gained and INMB

Across both models, the PinPoint test as a prioritization tool *marginally* improved the 10-year survival for cancer patients compared to standard care for all scenarios (see Tables 3 and 4). For prostate cancer patients, the PinPoint test led to a total LYG per patient ranging between 0.0000017 and 0.000052 depending on the scenario of interest. For bladder and kidney cancer, PinPoint yielded between 0.000021 and 0.001476 LYG per patient over standard care, depending on the scenario.

Implementing the PinPoint test as a triage and prioritization tool, however, resulted in a *marginal* reduction in the 10-year survival for cancer patients across both models due to a proportion of false-negative cases who initially were not urgently referred and then accessed secondary care after a 6-month delay. In the prostate cancer model, the PinPoint test resulted in a total of life years lost ranging between 0.000004 and 0.0000599 per patient depending on the scenario being evaluated. In the kidney/bladder cancer model, the PinPoint test as a triage and prioritization tool led to 0.0088 and 0.013 life years lost.

We observed larger changes in incremental life years for bladder/kidney cancer patients as opposed to prostate cancer ones, suggesting that the aggressiveness of the cancer impacts the detrimental effect of delayed diagnosis on long-term survival.

Assuming no difference in HRQoL, the PinPoint test implemented as a prioritization tool led to a negative overall INMB due to higher costs, despite a marginal improvement in LYG compared to standard care. In the prostate cancer model, the INMB ranged between −£89,100 and −£27,499 depending on the scenario; in the bladder/ kidney cancer model, the INMB for PinPoint varied between −£9,664 and −£27,635. Implementing the PinPoint test as a triage and prioritization tool, however, yielded a positive overall INMB due to its significant cost savings despite yielding marginal life years lost. In the prostate cancer model, the INMB ranged between £20,432 and £66,335, whereas in the bladder/kidney model, the INMB varied between £14,991 and £50,612.

These results should be interpreted with caution; the evidence used to estimate the life years lost is weak and multiple modeling assumptions have been made to arrive at these exploratory results.

## Discussion

We have developed two early economic models to explore the potential cost-effectiveness of the PinPoint test as a triage and/or prioritization tool for patients meeting the criteria of urgent referral for suspected urological cancer. The results suggest that, regardless of the use case, the PinPoint test could increase the proportion of individuals with cancer being seen within 2 weeks, while those without cancer would have to wait longer to be seen in secondary care. This result held regardless of the volume of referrals or the providers’ performance, although the benefits of the PinPoint test were greatest when a provider is struggling to meet the TWW target and a high volume of patients are being urgently referred.

If implemented solely as a prioritization tool, this benefit comes at an additional cost (£35 to £40 per patient). The acceptability of any additional cost, whether the PinPoint test could be used to relieve financial pressures, or the willingness to pay for individuals with cancer to be seen quicker, are currently unclear.

If implemented as a triage and prioritization tool, the PinPoint test has the potential to be cost saving. The shorter waiting times in this scenario do not only benefit those with cancer; taking individuals off the urgent suspected cancer pathway means that every urgently referred individual is seen quicker. However, a small proportion of individuals with cancer may experience a delay in diagnosis by being incorrectly classified as low risk and therefore not being urgently referred. It is difficult at this stage to estimate the cost, management, and health impact for this group of individuals (if any), as it will depend on the safety netting procedures put in place alongside the PinPoint test and the long-term consequence of any delay experienced. Further research is also needed to understand what happens to individuals receiving a true negative PinPoint test result. If a high proportion of these individuals are still referred down non-urgent pathways, then the estimated cost savings are likely to be smaller.

Based on highly uncertain estimates of the impact on life years, our exploratory analyses suggest that the PinPoint test is much more likely to be cost-effective if implemented as a triage and prioritization tool. The PinPoint test used as solely as a prioritization tool is unlikely to be cost-effective given the marginal improvement in survival and additional costs; the positive impact of earlier diagnosis for those with cancer on quality-of-life would have to be considerable for the PinPoint test to be cost-effective. However, if implemented as a triage and prioritization tool, the harms of the PinPoint test in terms of HRQoL would have to be considerable for the test to not be cost-effective, particularly as they apply to such a small patient subgroup (i.e., false-negative patients) and the test leads to substantial cost-savings. In addition, if individuals correctly classed as a “low risk” were found to experience delayed time-to-diagnosis for other conditions with a considerable detrimental effect on their health or HRQoL, PinPoint test might no longer be cost-effective.

### Evidence gaps

As an early economic modeling exercise, this analysis was not intended to provide conclusive evidence on the cost-effectiveness of the PinPoint test, but rather to highlight where the key gaps in the existing evidence lie.

Our modeling largely focuses on short-term outcomes because of the paucity of evidence on the impact of short-term delays to diagnosis on longer-term costs and health outcomes. The cost-effectiveness of the urgent suspected cancer referral pathway has never been evaluated, possibly for this reason. We are struggling to identify a realistic solution to this, as a clinical study sufficiently powered to demonstrate the marginal differences, if any, that a risk prediction tool like the PinPoint test would have on life years and long-term HRQoL is likely unfeasible.

There is ongoing an international debate about the feasibility of producing robust evidence of the long-term impact of earlier cancer diagnosis. Recent evidence questions whether earlier detection of cancer via screening actually saves lives (15) and, where evidence to demonstrate an all-cause or disease-specific mortality benefit is infeasible to generate, whether the use of surrogate outcomes (e.g. stage-shift or reduction in absolute incidence of late-stage cancer) is appropriate (16;17). Given the difficulties in estimating the potential benefits of early cancer diagnosis, Schwartzberg (18) conducted a modified Delphi panel with expert oncologists to estimate how long different cancers would take to progress from the beginning of one stage to the beginning of the next. For prostate cancer, the median number of years for each stage to progress ranged from 3 to 7 years. For bladder cancer, it ranged from <1 to 3 years, and for kidney cancer, it ranged from 2 to 5 years. If these figures are accurate, then a delay of a few weeks to diagnosis is unlikely to have long-term cost and health impacts (except perhaps for bladder cancer) and our shorter-term model horizon is sufficient.

Experiencing longer waiting times to be seen in secondary care, however, particularly in the context of patients who have clear and persistent symptoms (regardless of cancer status) is likely to cause anxiety. Measuring the impact of delays to diagnosis on short-term HRQoL would therefore be useful to ensure all relevant health benefits and harms for all patients are incorporated into future evaluations.

### Strengths and limitations

Our economic models offer a simple approach for evaluating the impact of the PinPoint test on waiting times and costs, given different volumes of referrals and the providers’ efficiency. This modeling approach could be easily applied to other suspected cancer pathways.

There are many limitations to the evidence base underpinning each model. As stated above, there is limited data on the health and cost consequences of relatively short-term delays to diagnosis for the majority of cancers. As part of an exploratory analysis, we used estimates from a UK-based modeling study (16) to explore the impact of the PinPoint test on life years. There is high uncertainty, however, in this evidence, and many modeling assumptions were applied, therefore results should be interpreted with caution.

There are also many unknown factors or uncertainties relating to the accuracy and implementation of the PinPoint test in real-world practice. A further prospective validation study is underway which includes all individuals referred on this pathway. This study will also produce data on the extent to which the PinPoint test correlates with the PSA test, as it will be important to understand what additional information the PinPoint test provides over the PSA test in terms of its ability to risk stratify individuals suspected of prostate cancer.

In addition to this, we did not explicitly capture the existing ability of standard care (if any) to prioritize patients for urgent referrals at the primary care level. For example, individuals with very high PSA levels would presumably be referred with more urgency for further investigations – although we are not aware of any formal quantitative process for prioritizing individuals (among those already receiving an urgent referral) based on PSA levels.

## Conclusions

Our early modeling suggests that the PinPoint test would improve waiting times for individuals with urological cancer referred down the urgent suspect cancer pathway. If used as a prioritization tool, implementing the PinPoint test will result in additional costs; whereas using PinPoint test as a triage and prioritization tool is highly likely to be cost saving and the harms associated with any “missed” cancers would have to be considerable for the PinPoint test to no longer be cost-effective. Although the PinPoint test could have an impact on longer-term costs and HRQoL, the differences are likely to be so marginal, that conducting a study sufficiently powered to produce evidence of these differences is likely infeasible. Exploring the impact of short-term delays on long-term quality-of-life would be helpful to explore how much money should be spent on resolving current delays to diagnosis.

## Supporting information

Cocco et al. supplementary material 1 Cocco et al. supplementary material

Cocco et al. supplementary material 2 Cocco et al. supplementary material

Cocco et al. supplementary material 3 Cocco et al. supplementary material

## Supplementary material

The supplementary material for this article can be found at https://doi.org/10.1017/S0266462324000023.
