# Supplementary material for: The National Health Service urgent cancer referral pathway for suspected urological cancers: early economic evaluation of a risk prediction test
Source: Int J Technol Assess Health Care. 2024 Jan 12;40(1):e9. doi: 10.1017/S0266462324000023 (PMC10859831; doi:10.1017/S0266462324000023)
Supplement: Cocco et al. supplementary material 1 — Cocco et al. supplementary material [file S0266462324000023sup001.docx]

# Supplementary File 1

## Model structure

**Supplementary Figure 1. 1 Simplified schematic of the expected implementation for the PinPoint test. Figure adapted from Smith, A.F. et al. (1)**


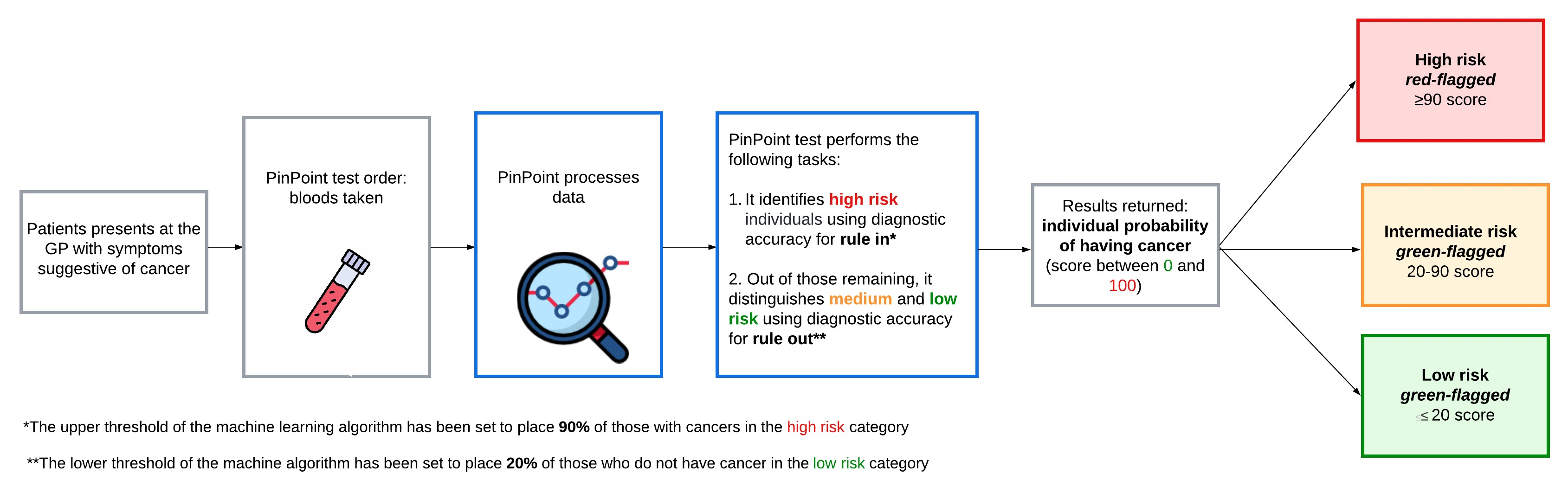


## Model parameters

**Supplementary Table 1. 1 Number of patients referred to the suspected urological cancer pathway and seen within two weeks between December 2021 and November 2022 across high, average, low volume of referrals providers, sorted by efficiency levels. Source: NHS Waiting Time Statistics [December 2021 to November 2022]**

|  | **Urological pathway** | | | | | | | | | | | | | | | | | |
| --- | --- | --- | --- | --- | --- | --- | --- | --- | --- | --- | --- | --- | --- | --- | --- | --- | --- | --- |
|  | **High volume referrals** | | | | | | **Average volume referrals** | | | | | | **Low volume referrals** | | | | | |
|  | *Top 25% performance* | | *Average performance* | | *Bottom 25% performance* | | *Top 25% performance* | | *Average performance* | | *Bottom 25% performance* | | *Top 25% performance* | | *Average performance* | | *Bottom 25% performance* | |
|  | **Total referrals (n)** | **Patients seen within 2 weeks (n)** | **Total referrals (n)** | **Patients seen within 2 weeks (n)** | **Total referrals (n)** | **Patients seen within 2 weeks (n)** | **Total referrals (n)** | **Patients seen within 2 weeks (n)** | **Total referrals (n)** | **Patients seen within 2 weeks (n)** | **Total referrals (n)** | **Patients seen within 2 weeks (n)** | **Total referrals (n)** | **Patients seen within 2 weeks (n)** | **Total referrals (n)** | **Patients seen within 2 weeks (n)** | **Total referrals (n)** | **Patients seen within 2 weeks (n)** |
| **Dec-21** | 307 | 300 | 282 | 256 | 272 | 193 | 150 | 149 | 158 | 149 | 153 | 119 | 82 | 81 | 89 | 84 | 90 | 71 |
| **Jan-22** | 208 | 201 | 242 | 219 | 258 | 166 | 124 | 122 | 139 | 124 | 141 | 87 | 64 | 63 | 75 | 69 | 83 | 61 |
| **Feb-22** | 266 | 260 | 248 | 233 | 287 | 222 | 138 | 135 | 142 | 131 | 150 | 98 | 80 | 79 | 85 | 81 | 78 | 66 |
| **Mar-22** | 311 | 303 | 329 | 304 | 307 | 220 | 175 | 173 | 177 | 164 | 193 | 145 | 104 | 103 | 104 | 97 | 101 | 82 |
| **Apr-22** | 239 | 231 | 270 | 239 | 260 | 177 | 149 | 144 | 152 | 131 | 158 | 105 | 72 | 71 | 95 | 89 | 80 | 65 |
| **May-22** | 267 | 259 | 268 | 245 | 314 | 213 | 162 | 160 | 149 | 139 | 166 | 124 | 74 | 74 | 93 | 88 | 89 | 71 |
| **Jun-22** | 251 | 241 | 252 | 223 | 263 | 183 | 146 | 143 | 148 | 134 | 151 | 110 | 81 | 80 | 94 | 87 | 78 | 55 |
| **Jul-22** | 284 | 275 | 284 | 259 | 236 | 175 | 155 | 152 | 152 | 141 | 156 | 113 | 96 | 94 | 95 | 87 | 88 | 63 |
| **Aug-22** | 277 | 269 | 295 | 270 | 250 | 192 | 152 | 151 | 162 | 150 | 154 | 110 | 88 | 87 | 92 | 85 | 98 | 77 |
| **Sep-22** | 281 | 273 | 309 | 279 | 258 | 189 | 163 | 162 | 164 | 152 | 168 | 125 | 92 | 90 | 102 | 92 | 100 | 71 |
| **Oct-22** | 310 | 302 | 318 | 292 | 312 | 201 | 186 | 183 | 184 | 169 | 181 | 130 | 101 | 99 | 114 | 108 | 95 | 67 |
| **Nov-22** | 364 | 356 | 328 | 295 | 339 | 219 | 183 | 181 | 181 | 163 | 180 | 106 | 96 | 95 | 121 | 112 | 92 | 73 |

**Supplementary Table 1. 2 Number of patients referred for prostate cancer and seen within two weeks between December 2021 and November 2022 across high, average, low volume of referrals providers, sorted by efficiency levels. Source: NHS Waiting Time Statistics [December 2021 to November 2022]**

|  | **Prostate cancer (75.91% urological pathway)** | | | | | | | | | | | | | | | | | |
| --- | --- | --- | --- | --- | --- | --- | --- | --- | --- | --- | --- | --- | --- | --- | --- | --- | --- | --- |
|  | **High volume referrals** | | | | | | **Average volume referrals** | | | | | | **Low volume referrals** | | | | | |
|  | *Top 25% performance* | | *Average performance* | | *Bottom 25% performance* | | *Top 25% performance* | | *Average performance* | | *Bottom 25% performance* | | *Top 25% performance* | | *Average performance* | | *Bottom 25% performance* | |
|  | **Total referrals (n)** | **Patients seen within 2 weeks (n)** | **Total referrals (n)** | **Patients seen within 2 weeks (n)** | **Total referrals (n)** | **Patients seen within 2 weeks (n)** | **Total referrals (n)** | **Patients seen within 2 weeks (n)** | **Total referrals (n)** | **Patients seen within 2 weeks (n)** | **Total referrals (n)** | **Patients seen within 2 weeks (n)** | **Total referrals (n)** | **Patients seen within 2 weeks (n)** | **Total referrals (n)** | **Patients seen within 2 weeks (n)** | **Total referrals (n)** | **Patients seen within 2 weeks (n)** |
| **Dec-21** | 233 | 228 | 214 | 195 | 206 | 146 | 114 | 113 | 120 | 113 | 116 | 90 | 62 | 62 | 68 | 64 | 69 | 54 |
| **Jan-22** | 158 | 152 | 184 | 166 | 196 | 126 | 94 | 93 | 105 | 94 | 107 | 66 | 49 | 48 | 57 | 53 | 63 | 46 |
| **Feb-22** | 202 | 197 | 188 | 177 | 218 | 168 | 105 | 102 | 108 | 99 | 114 | 74 | 61 | 60 | 65 | 61 | 59 | 50 |
| **Mar-22** | 236 | 230 | 250 | 231 | 233 | 167 | 133 | 131 | 134 | 124 | 147 | 110 | 79 | 78 | 79 | 74 | 77 | 62 |
| **Apr-22** | 181 | 175 | 205 | 181 | 197 | 134 | 113 | 109 | 115 | 99 | 120 | 80 | 55 | 54 | 72 | 67 | 61 | 49 |
| **May-22** | 203 | 197 | 203 | 186 | 238 | 162 | 123 | 122 | 113 | 106 | 126 | 94 | 56 | 56 | 70 | 66 | 67 | 54 |
| **Jun-22** | 190 | 183 | 192 | 170 | 200 | 139 | 111 | 109 | 112 | 102 | 114 | 84 | 62 | 61 | 71 | 66 | 59 | 42 |
| **Jul-22** | 215 | 209 | 216 | 196 | 179 | 132 | 117 | 116 | 115 | 107 | 118 | 86 | 73 | 72 | 72 | 66 | 67 | 48 |
| **Aug-22** | 210 | 205 | 224 | 205 | 189 | 146 | 116 | 115 | 123 | 114 | 117 | 84 | 67 | 66 | 70 | 64 | 74 | 58 |
| **Sep-22** | 213 | 207 | 235 | 212 | 196 | 144 | 124 | 123 | 125 | 115 | 127 | 95 | 69 | 68 | 77 | 70 | 76 | 54 |
| **Oct-22** | 235 | 229 | 242 | 221 | 237 | 152 | 141 | 139 | 139 | 128 | 137 | 99 | 76 | 75 | 86 | 82 | 72 | 51 |
| **Nov-22** | 276 | 270 | 249 | 224 | 257 | 167 | 139 | 137 | 137 | 124 | 137 | 81 | 73 | 72 | 92 | 85 | 70 | 56 |

**Supplementary Table 1. 3 Number of patients referred for bladder and kidney cancer seen within two weeks between December 2021 and November 2022 across high, average, low volume of referrals providers, sorted by efficiency levels. Source: NHS Waiting Time Statistics [December 2021 to November 2022]**

|  | **Bladder &kidney cancer (24.09% urological pathway)** | | | | | | | | | | | | | | | | | |
| --- | --- | --- | --- | --- | --- | --- | --- | --- | --- | --- | --- | --- | --- | --- | --- | --- | --- | --- |
|  | **High volume referrals** | | | | | | **Average volume referrals** | | | | | | **Low volume referrals** | | | | | |
|  | *Top 25% performance* | | *Average performance* | | *Bottom 25% performance* | | *Top 25% performance* | | *Average performance* | | *Bottom 25% performance* | | *Top 25% performance* | | *Average performance* | | *Bottom 25% performance* | |
|  | **Total referrals (n)** | **Patients seen within 2 weeks (n)** | **Total referrals (n)** | **Patients seen within 2 weeks (n)** | **Total referrals (n)** | **Patients seen within 2 weeks (n)** | **Total referrals (n)** | **Patients seen within 2 weeks (n)** | **Total referrals (n)** | **Patients seen within 2 weeks (n)** | **Total referrals (n)** | **Patients seen within 2 weeks (n)** | **Total referrals (n)** | **Patients seen within 2 weeks (n)** | **Total referrals (n)** | **Patients seen within 2 weeks (n)** | **Total referrals (n)** | **Patients seen within 2 weeks (n)** |
| **Dec-21** | 74 | 72 | 68 | 62 | 65 | 46 | 36 | 36 | 38 | 36 | 37 | 29 | 20 | 20 | 21 | 20 | 22 | 17 |
| **Jan-22** | 50 | 48 | 58 | 53 | 62 | 40 | 30 | 29 | 33 | 30 | 34 | 21 | 15 | 15 | 18 | 17 | 20 | 15 |
| **Feb-22** | 64 | 63 | 60 | 56 | 69 | 53 | 33 | 32 | 34 | 31 | 36 | 24 | 19 | 19 | 21 | 19 | 19 | 16 |
| **Mar-22** | 75 | 73 | 79 | 73 | 74 | 53 | 42 | 42 | 43 | 39 | 46 | 35 | 25 | 25 | 25 | 23 | 24 | 20 |
| **Apr-22** | 58 | 56 | 65 | 57 | 63 | 43 | 36 | 35 | 37 | 31 | 38 | 25 | 17 | 17 | 23 | 21 | 19 | 16 |
| **May-22** | 64 | 62 | 65 | 59 | 76 | 51 | 39 | 39 | 36 | 34 | 40 | 30 | 18 | 18 | 22 | 21 | 21 | 17 |
| **Jun-22** | 60 | 58 | 61 | 54 | 63 | 44 | 35 | 34 | 36 | 32 | 36 | 27 | 20 | 19 | 23 | 21 | 19 | 13 |
| **Jul-22** | 68 | 66 | 68 | 62 | 57 | 42 | 37 | 37 | 37 | 34 | 38 | 27 | 23 | 23 | 23 | 21 | 21 | 15 |
| **Aug-22** | 67 | 65 | 71 | 65 | 60 | 46 | 37 | 36 | 39 | 36 | 37 | 27 | 21 | 21 | 22 | 20 | 24 | 19 |
| **Sep-22** | 68 | 66 | 74 | 67 | 62 | 46 | 39 | 39 | 40 | 37 | 40 | 30 | 22 | 22 | 25 | 22 | 24 | 17 |
| **Oct-22** | 75 | 73 | 77 | 70 | 75 | 48 | 45 | 44 | 44 | 41 | 44 | 31 | 24 | 24 | 27 | 26 | 23 | 16 |
| **Nov-22** | 88 | 86 | 79 | 71 | 82 | 53 | 44 | 43 | 43 | 39 | 43 | 26 | 23 | 23 | 29 | 27 | 22 | 18 |

**Supplementary Table 1. 4 Number and proportion of suspected urological cancer patients seen after two weeks between December 2021 and November 2022 across high volume of referrals providers, sorted by efficiency levels. Source: NHS Waiting Time Statistics [December 2021 to November 2022]**

|  | **Urological pathway** | | | | | | | | | | | | | | |
| --- | --- | --- | --- | --- | --- | --- | --- | --- | --- | --- | --- | --- | --- | --- | --- |
|  | **High volume referrals** | | | | | | | | | | | | | | |
|  | *Top 25% performance* | | | | | *Average performance* | | | | | *Bottom 25% performance* | | | | |
|  | **Average patients seen after two weeks** | **Average patients seen in 15 to 16 days (%)** | **Average patients seen in 17 to 21 days (%)** | **Average patients seen in 22 to 27 days (%)** | **Average patients seen in after 28 days (%)** | **Average patients seen after two weeks** | **Average patients seen in 15 to 16 days (%)** | **Average patients seen in 17 to 21 days (%)** | **Average patients seen in 22 to 27 days (%)** | **Average patients seen in after 28 days (%)** | **Average patients seen after two weeks** | **Average patients seen in 15 to 16 days (%)** | **Average patients seen in 17 to 21 days (%)** | **Average patients seen in 22 to 27 days (%)** | **Average patients seen in after 28 days (%)** |
| **Dec-21** | 6 | 2.3 (36.21%) | 2 (31.03%) | 1.6 (31.03%) | 0.6 (8.62%) | 25 | 9.8 (38.92%) | 9 (35.86%) | 3.5 (13.87%) | 2.9 (11.35%) | 79 | 24 (30.42%) | 28.2 (35.77%) | 17.4 (22.11%) | 9.2 (11.69%) |
| **Jan-22** | 7 | 1.3 (18.84%) | 2.9 (42.03%) | 1.6 (23.19%) | 1.1 (15.94%) | 24 | 6.5 (27.27%) | 8.9 (37.63%) | 4.7 (19.87%) | 3.6 (15.22%) | 93 | 18.3 (19.76%) | 26.5 (28.62%) | 30.7 (33.15%) | 17.1 (18.47%) |
| **Feb-22** | 6 | 2.2 (34.92%) | 2.6 (41.27%) | 0.5 (7.94%) | 1 (15.87%) | 15 | 5.8 (38.21%) | 5.3 (34.88%) | 2.8 (18.27%) | 1.3 (8.64%) | 65 | 19.1 (29.34%) | 26.8 (41.17%) | 11 (16.9%) | 8.2 (12.6%) |
| **Mar-22** | 8 | 2.8 (34.15%) | 3.4 (41.46%) | 1.2 (14.63%) | 0.8 (9.76%) | 25 | 10.1 (39.49%) | 10.1 (39.69%) | 3.5 (13.56%) | 1.9 (7.27%) | 87 | 27.2 (31.23%) | 29.2 (33.52%) | 18.1 (20.78%) | 12.6 (14.47%) |
| **Apr-22** | 8 | 2.4 (28.92%) | 3.6 (43.37%) | 1.3 (15.66%) | 1 (12.05%) | 31 | 9.5 (30.7%) | 11.3 (36.26%) | 6.5 (20.76%) | 3.8 (12.28%) | 83 | 23.6 (28.47%) | 30.9 (37.27%) | 19.1 (23.04%) | 9.3 (11.22%) |
| **May-22** | 8 | 2.4 (31.17%) | 3 (38.96%) | 1.7 (22.08%) | 0.6 (7.79%) | 23 | 6.6 (28.45%) | 9.4 (40.82%) | 4.3 (18.56%) | 2.8 (12.16%) | 101 | 19.6 (19.48%) | 32.2 (32.01%) | 27.8 (27.63%) | 21 (20.87%) |
| **Jun-22** | 9 | 3.5 (37.63%) | 4.4 (47.31%) | 0.7 (7.53%) | 0.7 (7.53%) | 29 | 11.2 (38.75%) | 10.8 (37.11%) | 4 (13.79%) | 3 (10.34%) | 81 | 25.6 (31.76%) | 25.6 (31.76%) | 13.3 (16.5%) | 16.1 (19.98%) |
| **Jul-22** | 8 | 2.3 (28.05%) | 3.4 (41.46%) | 1.7 (20.73%) | 0.8 (9.76%) | 25 | 7.6 (29.67%) | 11.9 (46.56%) | 4.6 (17.88%) | 1.5 (5.89%) | 61 | 17.1 (27.9%) | 20.6 (33.61%) | 13.5 (22.02%) | 10.1 (16.48%) |
| **Aug-22** | 8 | 2.4 (31.58%) | 2.8 (36.84%) | 1.6 (21.05%) | 0.8 (10.53%) | 24 | 7.9 (32.24%) | 9.5 (39.01%) | 4.8 (19.51%) | 2.3 (9.24%) | 58 | 15.3 (26.61%) | 17.7 (30.78%) | 11.7 (20.35%) | 12.8 (22.26%) |
| **Sep-22** | 8 | 2.7 (32.53%) | 3.3 (39.76%) | 1.6 (19.28%) | 0.7 (8.43%) | 30 | 9.6 (31.78%) | 11.6 (38.44%) | 5 (16.64%) | 4 (13.14%) | 69 | 17.1 (24.82%) | 26.3 (38.17%) | 15 (21.77%) | 10.5 (15.24%) |
| **Oct-22** | 8 | 2.7 (33.75%) | 3.1 (38.75%) | 1.5 (18.75%) | 0.7 (8.75%) | 27 | 6.5 (24.48%) | 11.5 (43.13%) | 5.6 (21.09%) | 3 (11.3%) | 112 | 22 (19.7%) | 39.2 (35.09%) | 33.1 (29.63%) | 17.4 (15.58%) |
| **Nov-22** | 9 | 2.5 (29.41%) | 3.3 (38.82%) | 2.4 (28.24%) | 0.3 (3.53%) | 33 | 9.9 (29.49%) | 13 (38.92%) | 5.7 (16.92%) | 4.9 (14.67%) | 120 | 20.3 (16.94%) | 37.4 (31.22%) | 27.6 (23.04%) | 34.5 (28.8%) |

**Supplementary Table 1. 5 Number and proportion of suspected urological cancer patients seen after two weeks between December 2021 and November 2022 across average volume of referrals providers, sorted by efficiency levels. Source: NHS Waiting Time Statistics [December 2021 to November 2022]**

|  | **Urological pathway** | | | | | | | | | | | | | | |
| --- | --- | --- | --- | --- | --- | --- | --- | --- | --- | --- | --- | --- | --- | --- | --- |
|  | **Average volume referrals** | | | | | | | | | | | | | | |
|  | *Top 25% performance* | | | | | *Average performance* | | | | | *Bottom 25% performance* | | | | |
|  | **Average patients seen after two weeks** | **Average patients seen in 15 to 16 days (%)** | **Average patients seen in 17 to 21 days (%)** | **Average patients seen in 22 to 27 days (%)** | **Average patients seen in after 28 days (%)** | **Average patients seen after two weeks** | **Average patients seen in 15 to 16 days (%)** | **Average patients seen in 17 to 21 days (%)** | **Average patients seen in 22 to 27 days (%)** | **Average patients seen in after 28 days (%)** | **Average patients seen after two weeks** | **Average patients seen in 15 to 16 days (%)** | **Average patients seen in 17 to 21 days (%)** | **Average patients seen in 22 to 27 days (%)** | **Average patients seen in after 28 days (%)** |
| **Dec-21** | 1 | 0.1 (12.5%) | 0.7 (62.5%) | 0.3 (25%) | 0 (0%) | 8 | 2.7 (33.06%) | 3.1 (37.9%) | 1.7 (20.16%) | 0.7 (8.87%) | 34 | 9 (26.58%) | 16 (47.26%) | 7 (20.68%) | 1.9 (5.49%) |
| **Jan-22** | 2 | 0.6 (30.77%) | 0.9 (46.15%) | 0.1 (7.69%) | 0.3 (15.38%) | 14 | 3.9 (27.27%) | 6.9 (48.05%) | 2.3 (15.58%) | 1.3 (9.09%) | 53 | 10.3 (19.3%) | 23.6 (44.24%) | 14.4 (27.08%) | 5 (9.38%) |
| **Feb-22** | 3 | 0.9 (27.27%) | 2.1 (68.18%) | 0 (0%) | 0.1 (4.55%) | 11 | 4.3 (37.79%) | 4.6 (40.12%) | 1.6 (13.95%) | 0.9 (8.14%) | 53 | 21.3 (40.27%) | 19.4 (36.76%) | 8 (15.14%) | 4.1 (7.84%) |
| **Mar-22** | 2 | 0.4 (25%) | 0.9 (50%) | 0.3 (16.67%) | 0.1 (8.33%) | 13 | 5.1 (38.31%) | 4.5 (33.83%) | 2.9 (21.39%) | 0.9 (6.47%) | 48 | 14.1 (29.38%) | 16.4 (34.12%) | 9.3 (19.29%) | 8.3 (17.21%) |
| **Apr-22** | 5 | 1.8 (37.5%) | 1.4 (29.17%) | 0.8 (16.67%) | 0.8 (16.67%) | 21 | 10.6 (50%) | 6.9 (32.68%) | 2.8 (13.39%) | 0.8 (3.94%) | 53 | 11.2 (21.13%) | 14.6 (27.55%) | 6.6 (12.45%) | 20.6 (38.87%) |
| **May-22** | 2 | 0.3 (15.38%) | 1 (53.85%) | 0.4 (23.08%) | 0.1 (7.69%) | 10 | 2 (20.29%) | 4.4 (44.93%) | 2 (20.29%) | 1.4 (14.49%) | 43 | 14.7 (34.56%) | 19 (44.63%) | 6.3 (14.77%) | 2.6 (6.04%) |
| **Jun-22** | 3 | 0.8 (31.25%) | 1.2 (43.75%) | 0.3 (12.5%) | 0.3 (12.5%) | 13 | 4.8 (36.48%) | 4.6 (34.59%) | 2.7 (20.13%) | 1.2 (8.81%) | 41 | 7.8 (19.26%) | 15.3 (37.7%) | 7.3 (18.03%) | 10.2 (25%) |
| **Jul-22** | 2 | 0.4 (17.65%) | 1.3 (52.94%) | 0.7 (29.41%) | 0 (0%) | 11 | 2.2 (20%) | 4.8 (44.32%) | 2.5 (23.24%) | 1.4 (12.43%) | 43 | 12.7 (29.87%) | 16.4 (38.59%) | 9.6 (22.48%) | 3.9 (9.06%) |
| **Aug-22** | 2 | 0.7 (42.86%) | 0.7 (42.86%) | 0.1 (7.14%) | 0.1 (7.14%) | 11 | 2.9 (25.68%) | 4.1 (36.94%) | 2.5 (22.07%) | 1.7 (15.32%) | 44 | 11.6 (26.26%) | 15.4 (35.1%) | 9.1 (20.71%) | 7.9 (17.93%) |
| **Sep-22** | 1 | 0.2 (18.18%) | 0.7 (54.55%) | 0.2 (18.18%) | 0.1 (9.09%) | 12 | 3.2 (26.41%) | 5 (41.13%) | 2.8 (22.94%) | 1.2 (9.52%) | 43 | 13.2 (30.59%) | 18 (41.65%) | 9.3 (21.59%) | 2.7 (6.17%) |
| **Oct-22** | 3 | 0.6 (55.56%) | 1.7 (166.67%) | 0.7 (66.67%) | 0.4 (44.44%) | 15 | 4 (27.05%) | 6.5 (44.13%) | 3.4 (22.78%) | 0.9 (6.05%) | 51 | 14.9 (29.19%) | 20.2 (39.65%) | 11.1 (21.79%) | 4.8 (9.37%) |
| **Nov-22** | 181 | 1.1 (50%) | 0.8 (36.36%) | 0.2 (9.09%) | 0.1 (4.55%) | 163 | 5.6 (31.62%) | 6.6 (37.61%) | 3.6 (20.23%) | 1.9 (10.54%) | 106 | 17.7 (23.98%) | 28 (37.94%) | 18.3 (24.8%) | 9.8 (13.28%) |

**Supplementary Table 1. 6** **Number and proportion of suspected urological cancer patients seen after two weeks between December 2021 and November 2022 across low volume of referrals providers, sorted by efficiency levels. Source: NHS Waiting Time Statistics [December 2021 to November 2022]**

|  | **Urological pathway** | | | | | | | | | | | | | | |
| --- | --- | --- | --- | --- | --- | --- | --- | --- | --- | --- | --- | --- | --- | --- | --- |
|  | **Low volume referrals** | | | | | | | | | | | | | | |
|  | *Top 25% performance* | | | | | *Average performance* | | | | | *Bottom 25% performance* | | | | |
|  | **Average patients seen after two weeks** | **Average patients seen in 15 to 16 days (%)** | **Average patients seen in 17 to 21 days (%)** | **Average patients seen in 22 to 27 days (%)** | **Average patients seen in after 28 days (%)** | **Average patients seen after two weeks** | **Average patients seen in 15 to 16 days (%)** | **Average patients seen in 17 to 21 days (%)** | **Average patients seen in 22 to 27 days (%)** | **Average patients seen in after 28 days (%)** | **Average patients seen after two weeks** | **Average patients seen in 15 to 16 days (%)** | **Average patients seen in 17 to 21 days (%)** | **Average patients seen in 22 to 27 days (%)** | **Average patients seen in after 28 days (%)** |
| **Dec-21** | 1 | 0.2 (28.57%) | 0.3 (42.86%) | 0.1 (14.29%) | 0.1 (14.29%) | 5 | 1.9 (39.36%) | 1.5 (29.79%) | 1.2 (24.47%) | 0.3 (6.38%) | 20 | 6.8 (34.69%) | 5.4 (27.55%) | 3.9 (19.9%) | 3.5 (17.86%) |
| **Jan-22** | 1 | 0.3 (37.5%) | 0.5 (62.5%) | 0 (0%) | 0 (0%) | 5 | 1.8 (34.31%) | 1.8 (34.31%) | 0.9 (16.67%) | 0.8 (14.71%) | 22 | 3.7 (16.97%) | 9.4 (43.12%) | 3.7 (16.97%) | 5 (22.94%) |
| **Feb-22** | 1 | 0.3 (50%) | 0.1 (16.67%) | 0.1 (16.67%) | 0.1 (16.67%) | 5 | 1.7 (36.26%) | 1.5 (32.97%) | 0.7 (15.38%) | 0.7 (15.38%) | 12 | 2.6 (21.85%) | 4.7 (39.5%) | 1.9 (15.97%) | 2.7 (22.69%) |
| **Mar-22** | 1 | 0.4 (40%) | 0.6 (60%) | 0 (0%) | 0 (0%) | 7 | 2.1 (31.34%) | 2.7 (40.3%) | 1.3 (19.4%) | 0.6 (8.96%) | 20 | 7.6 (38.97%) | 7.7 (39.49%) | 3.5 (17.95%) | 0.7 (3.59%) |
| **Apr-22** | 1 | 0.2 (22.22%) | 0.6 (66.67%) | 0.1 (11.11%) | 0 (0%) | 7 | 2.2 (31.39%) | 2.6 (37.23%) | 1.3 (18.98%) | 0.9 (12.41%) | 16 | 5.4 (34.18%) | 7.6 (48.1%) | 1.8 (11.39%) | 1 (6.33%) |
| **May-22** | 0 | 0.1 (100%) | 0 (0%) | 0 (0%) | 0 (0%) | 5 | 1.8 (36%) | 2.2 (43%) | 0.5 (9%) | 0.6 (12%) | 18 | 4.1 (23.43%) | 7.8 (44.57%) | 2.3 (13.14%) | 3.3 (18.86%) |
| **Jun-22** | 1 | 0.6 (54.55%) | 0.4 (36.36%) | 0 (0%) | 0.1 (9.09%) | 7 | 2.2 (32.33%) | 3 (45.11%) | 0.8 (12.03%) | 0.7 (10.53%) | 23 | 9.2 (40.71%) | 8.2 (36.28%) | 3.5 (15.49%) | 1.7 (7.52%) |
| **Jul-22** | 2 | 0.5 (29.41%) | 0.9 (52.94%) | 0.3 (17.65%) | 0 (0%) | 8 | 2.8 (35.48%) | 3.1 (39.35%) | 1.3 (16.13%) | 0.7 (9.03%) | 25 | 5.1 (20.56%) | 10.4 (41.94%) | 5.2 (20.97%) | 4.1 (16.53%) |
| **Aug-22** | 1 | 0.3 (25%) | 0.5 (41.67%) | 0.3 (25%) | 0.1 (8.33%) | 7 | 2.3 (31.69%) | 2.8 (39.44%) | 1.5 (21.13%) | 0.6 (7.75%) | 21 | 5.4 (25.71%) | 7.8 (37.14%) | 3.7 (17.62%) | 4.1 (19.52%) |
| **Sep-22** | 2 | 0.5 (27.78%) | 0.8 (44.44%) | 0.1 (5.56%) | 0.4 (22.22%) | 23 | 3.3 (33.33%) | 3.6 (36.23%) | 2 (19.81%) | 1 (10.63%) | 29 | 9.9 (33.9%) | 9.7 (33.22%) | 5.8 (19.86%) | 3.8 (13.01%) |
| **Oct-22** | 2 | 0.5 (33.33%) | 0.4 (26.67%) | 0.3 (20%) | 0.3 (20%) | 6 | 1.4 (25.47%) | 2.5 (44.34%) | 1 (17.92%) | 0.7 (12.26%) | 28 | 7.4 (26.33%) | 11.7 (41.64%) | 6.6 (23.49%) | 2.4 (8.54%) |
| **Nov-22** | 1 | 0.2 (33.33%) | 0.3 (50%) | 0.1 (16.67%) | 0 (0%) | 8 | 2.4 (28.74%) | 2.7 (31.74%) | 2.5 (29.94%) | 0.8 (9.58%) | 19 | 5.9 (31.05%) | 8 (42.11%) | 4.3 (22.63%) | 0.8 (4.21%) |

**Supplementary Table 1. 7 Age-stratified life years lost per patient seen in secondary care with a 2, 4 and 6-month delay from GP referral. Figure adapted from Sud, A. et al. (2)**

## Model analysis

### Deterministic sensitivity analysis

**Supplementary Figure 1. 2 Assessment of how many model replications are required to stabilise the life years lost in the prostate cancer model– standard care, providers with average volume of referrals and average performance levels**

**Supplementary Figure 1. 3 Assessment of how many model replications are required to stabilise the life years lost in the bladder and kidney cancer model– standard care, providers with average volume of referrals and average performance levels**

### Probabilistic sensitivity analysis

For each scenario a probabilistic sensitivity analysis (PSA) was conducted running 1,200 replications, with each run using a different parameter set drawn from probabilistic distributions We applied probabilistic distributions to key parameters of interest (i.e. diagnostic accuracy of PinPoint test, disease prevalence, costs – see Supplementary Table 8) to minimise the impact of second-order uncertainty. We selected gamma distributions for cost parameters, and beta distributions for diagnostic accuracy estimates and disease prevalence – in the context of parameters where the standard error was unknown, we assumed a standard deviation equal to 0.1 of the mean value.

**Supplementary Table 1. 8 Deterministic estimate, chosen probabilistic distribution and related shape variables for each model parameter varied during the probabilistic sensitivity analysis.**

| **Parameters common to the prostate and kidney/renal bladder cancer models** | | | | |
| --- | --- | --- | --- | --- |
| **Parameter** | **Deterministic estimate** | **Probabilistic distribution** | **Probabilistic distribution shape parameters** | **Notes for estimation probabilistic distribution parameters** |
| Urological cancer prevalence | 16.9% | Beta | Alpha: 0.06834191  Beta: 0.33604809 | Unknown standard error (SE). Assumed SE to be equal 10% of mean estimate |
| Diagnostic sensitivity PinPoint test [rule in] – urological pathway | 90.04% | Beta | Alpha: 7.771684754  Beta: 0.859684364 | 95% confidence interval (CI): 0.8817-0.9182. Sample size: 4206. (3) |
| Diagnostic specificity PinPoint test [rule in] – urological pathway | 35.48% | Beta | Alpha: 9.263972527  Beta: 16.84643482 | 95% CI: 0.3379-0.371. Sample size: 4206. (3) |
| Diagnostic sensitivity PinPoint test [rule out] – urological pathway | 96.81% | Beta | Alpha: 4.43269206  Beta: 0.146062263 | 95% CI: 0.9568-0.9785. Sample size: 4206. (3) |
| Diagnostic specificity PinPoint test [rule out] – urological pathway | 20.02% | Beta | Alpha: 4.336247028  Beta: 17.32332854 | 95% CI: 0.1864-0.2141. Sample size: 4206. (3) |
| Cost of GP consultation | £45.44 | Gamma | Alpha: 20647.936  Beta: 0.002200704 | Unknown SE. Assumed SE to be equal 10% of mean estimate |
| Cost of multidisciplinary cancer team meeting | £165.82 | Gamma | Alpha: 274962.724  Beta: 0.000603064 | Unknown SE. Assumed SE to be equal 10% of mean estimate |
| **Parameters specific to the prostate cancer model** | | | | |
| Cost of PSA testing | £5.68 | Gamma | Alpha: 322.624  Beta: 0.017605634 | Unknown SE. Assumed SE to be equal 10% of mean estimate |
| Cost of PinPoint test | £35.17 | Gamma | Alpha: 12369.289  Beta: 0.002843332 | Unknown SE. Assumed SE to be equal 10% of mean estimate |
| Cost of triage | £29.13 | Gamma | Alpha: 8485.569  Beta: 0.003432887 | Unknown SE. Assumed SE to be equal 10% of mean estimate |
| Cost of mpMRI | £341.86 | Gamma | Alpha: 1168682.596  Beta: 0.000292517 | Unknown SE. Assumed SE to be equal 10% of mean estimate |
| Cost of TRUS biopsy | £927.91 | Gamma | Alpha: 8610169.681  Beta: 0.000107769 | Unknown SE. Assumed SE to be equal 10% of mean estimate |
| **Parameters specific to the bladder/kidney cancer model** | | | | |
| Cost of PinPoint test | £39.17 | Gamma | Alpha: 15342.889  Beta: 0.002552974 | Unknown SE. Assumed SE to be equal 10% of mean estimate |
| Cost of cystoscopy | £306.67 | Gamma | Alpha: 940464.889  Beta: 0.000326083 | Unknown SE. Assumed SE to be equal 10% of mean estimate |
| Cost of Xray | £51.48 | Gamma | Alpha: 26501.904  Beta: 0.001942502 | Unknown SE. Assumed SE to be equal 10% of mean estimate |
| Cost of ultrasound | £78.35 | Gamma | Alpha: 61387.225  Beta: 0.001276324 | Unknown SE. Assumed SE to be equal 10% of mean estimate |

1. Smith, A.F., Frempong, S.N., Sharma, N., Neal, R.D., Hick, L. and Shinkins, B. An exploratory assessment of the impact of a novel risk assessment test on breast cancer clinic waiting times and workflow: a discrete event simulation model. *BMC Health Services Research.* 2022, **22**(1), p.1301.

2. Sud, A., Torr, B., Jones, M.E., Broggio, J., Scott, S., Loveday, C., Garrett, A., Gronthoud, F., Nicol, D.L., Jhanji, S., Boyce, S.A., Williams, M., Riboli, E., Muller, D.C., Kipps, E., Larkin, J., Navani, N., Swanton, C., Lyratzopoulos, G., McFerran, E., Lawler, M., Houlston, R. and Turnbull, C. Effect of delays in the 2-week-wait cancer referral pathway during the COVID-19 pandemic on cancer survival in the UK: a modelling study. *The Lancet Oncology.* 2020, **21**(8), pp.1035-1044.

3. Savage, R., Messenger, M., Neal, R.D., Ferguson, R., Johnston, C., Lloyd, K.L., Neal, M.D., Sansom, N., Selby, P., Sharma, N., Shinkins, B., Skinner, J.R., Tully, G., Duffy, S. and Hall, G. Development and validation of multivariable machine learning algorithms to predict risk of cancer in symptomatic patients referred urgently from primary care: a diagnostic accuracy study. *BMJ Open.* 2022, **12**(4), p.e053590.
