# Supplementary material for: The National Health Service urgent cancer referral pathway for suspected urological cancers: early economic evaluation of a risk prediction test
Source: Int J Technol Assess Health Care. 2024 Jan 12;40(1):e9. doi: 10.1017/S0266462324000023 (PMC10859831; doi:10.1017/S0266462324000023)
Supplement: Cocco et al. supplementary material 2 — Cocco et al. supplementary material [file S0266462324000023sup002.docx]

# Supplementary File 2

The models were developed in accordance with the Consolidated Health Economic Evaluation Reporting Standards checklist (CHEERS, 2022) [1] and the Strengthening The Reporting of Empirical Simulation Studies guidelines [2].

**Supplementary table 2. 1 CHEERS checklist**

| **Topic** | **No.** | **Item** | **Location where item is reported** |
| --- | --- | --- | --- |
| **Title** |  |  |  |
|  | 1 | Identify the study as an economic evaluation and specify the interventions being compared. | Manuscript, Title |
| **Abstract** |  |  |  |
|  | 2 | Provide a structured summary that highlights context, key methods, results, and alternative analyses. | Manuscript, Abstract |
| **Introduction** |  |  |  |
| **Background and objectives** | 3 | Give the context for the study, the study question, and its practical relevance for decision making in policy or practice. | Manuscript, Introduction. |
| **Methods** |  |  |  |
| **Health economic analysis plan** | 4 | Indicate whether a health economic analysis plan was developed and where available. | Not applicable |
| **Study population** | 5 | Describe characteristics of the study population (such as age range, demographics, socioeconomic, or clinical characteristics). | Manuscript, Model structure |
| **Setting and location** | 6 | Provide relevant contextual information that may influence findings. | Manuscript, Model structure |
| **Comparators** | 7 | Describe the interventions or strategies being compared and why chosen. | Manuscript, Model structure |
| **Perspective** | 8 | State the perspective(s) adopted by the study and why chosen. | Manuscript, Model analysis |
| **Time horizon** | 9 | State the time horizon for the study and why appropriate. | Manuscript, Model analysis |
| **Discount rate** | 10 | Report the discount rate(s) and reason chosen. | Manuscript, Model analysis. |
| **Selection of outcomes** | 11 | Describe what outcomes were used as the measure(s) of benefit(s) and harm(s). | Manuscript, Model analysis. |
| **Measurement of outcomes** | 12 | Describe how outcomes used to capture benefit(s) and harm(s) were measured. | Manuscript, Model analysis. |
| **Valuation of outcomes** | 13 | Describe the population and methods used to measure and value outcomes. | Not applicable |
| **Measurement and valuation of resources and costs** | 14 | Describe how costs were valued. | Manuscript, Model parameters. |
| **Currency, price date, and conversion** | 15 | Report the dates of the estimated resource quantities and unit costs, plus the currency and year of conversion. | Manuscript, Model parameters. |
| **Rationale and description of model** | 16 | If modelling is used, describe in detail and why used. Report if the model is publicly available and where it can be accessed. | Manuscript, Model structure, Supplementary File 2 |
| **Analytics and assumptions** | 17 | Describe any methods for analysing or statistically transforming data, any extrapolation methods, and approaches for validating any model used. | Manuscript, Model assumptions. |
| **Characterising heterogeneity** | 18 | Describe any methods used for estimating how the results of the study vary for subgroups. | Not applicable |
| **Characterising distributional effects** | 19 | Describe how impacts are distributed across different individuals or adjustments made to reflect priority populations. | Not applicable |
| **Characterising uncertainty** | 20 | Describe methods to characterise any sources of uncertainty in the analysis. | Manuscript, Model analysis |
| **Approach to engagement with patients and others affected by the study** | 21 | Describe any approaches to engage patients or service recipients, the general public, communities, or stakeholders (such as clinicians or payers) in the design of the study. | Manuscript, Model structure. |
| **Results** |  |  |  |
| **Study parameters** | 22 | Report all analytic inputs (such as values, ranges, references) including uncertainty or distributional assumptions. | Manuscript, Table 1 |
| **Summary of main results** | 23 | Report the mean values for the main categories of costs and outcomes of interest and summarise them in the most appropriate overall measure. | Manuscript, Results |
| **Effect of uncertainty** | 24 | Describe how uncertainty about analytic judgments, inputs, or projections affect findings. Report the effect of choice of discount rate and time horizon, if applicable. | Manuscript, Results |
| **Effect of engagement with patients and others affected by the study** | 25 | Report on any difference patient/service recipient, general public, community, or stakeholder involvement made to the approach or findings of the study | Not applicable |
| **Discussion** |  |  |  |
| **Study findings, limitations, generalisability, and current knowledge** | 26 | Report key findings, limitations, ethical or equity considerations not captured, and how these could affect patients, policy, or practice. | Manuscript, Discussion |
| **Other relevant information** |  |  |  |
| **Source of funding** | 27 | Describe how the study was funded and any role of the funder in the identification, design, conduct, and reporting of the analysis | Page iii |
| **Conflicts of interest** | 28 | Report authors conflicts of interest according to journal or International Committee of Medical Journal Editors requirements. | Page iii |

**Supplementary table 2. 2 STRESS-DES checklist**

| **Section/Subsection** | **Item** | **Details/location in Manuscript where details are reported** | |
| --- | --- | --- | --- |
| 1. **Objectives** |  | | |
| Purpose of the model | 1.1 | In collaboration with the Leeds Teaching Hospitals NHS Trust and the University of Leeds, PinPoint Data Science Ltd have developed a multivariable machine learning algorithm, hereafter referred to as the PinPoint test, to predict risk of cancer in symptomatic patients urgently referred from primary care. The test has been designed to determine patients’ risk of cancer based on several routine blood tests (including haematological, biochemical and tumour markers), producing a calibrated risk probability of cancer ranging between 0 and 1 (with higher values indicating a higher risk of having cancer). The PinPoint test has been developed to (1) to identify and prioritise high risk individuals (i.e. fast-tracking urgent referrals for these individuals) [‘prioritisation’ use case]; and (2) to identify individuals who can be safely removed from the two-week waiting cancer pathways and prioritise high risk individuals for urgent referrals [‘triage and prioritisation’ use case] based on a blood test.  The aim of the urological models presented in the manuscript was to explore the potential cost-effectiveness of the PinPoint test compared to usual care in primary and secondary care from a UK NHS perspective for patients with urgent suspected urological cancer, including (i) prostate cancer; and (ii) bladder and kidney cancer.  The models focused on the potential application of PinPoint test as (i) a prioritisation tool to identify patients with high risk of having cancer who should be fast-tracked for urgent referrals in secondary care for further investigations; and (ii) triage patients with low risk of having cancer and prioritise patients with high risk of having cancer for urgent referral in secondary care. | |
| Model Outputs | 1.2 | The urological models evaluated three primary outcomes, including: (i) referral patterns (i.e. number of patients referred and not referred, number of patients seen within and two weeks, number of patients seen after 28 days); and (iii) total costs. | |
| Experimentation Aims | 1.3 | The experimental aims of these models were two-fold, including: (i) to determine the impact of the PinPoint test used as a triage and/or prioritisation tool on the ability of secondary care cancer clinics to see more patients in a more timely manner upon GP referral; (ii) to evaluate the cost-effectiveness of PinPoint test compared to usual care.  The findings of these early economic models should be considered exploratory only as the early development stages of the PinPoint test. | |
| 1. **Logic** |  | | |
| Base model overview diagram | 2.1 | The structure of the urological models is presented in **Figure 1**.  It was decided to develop a discrete event simulation (DES) model due to the primary advantages of DES modelling, including: (i) the ability to accurately measure the timing of events, and their associated costs and outcomes; (iii) the ability to capture a sequence of hospital processes patients suspected with urological cancer undergo; and (iv) the ability to capture capacity constraints and queues in the system (i.e. limited capacity to see patients within two weeks upon GP referral) [3-5].  Both models track the flow of patients suspected with urological cancer from GP consultation in primary care up to diagnosis in secondary care. All patients with symptoms indicative of urological cancer are urgently referred to secondary care for further investigations. After urgent referral to secondary care, all patients wait a minimum of 13 days due to (i) logistical reasons (e.g. booking, phone calls) and (ii) based on the NHS England Waiting Time Statistics, it is unknown exactly the day within two weeks patients are actually seen in secondary care. The secondary care clinic has a limited capacity to see patients within two weeks upon referral – depending on the number of patients referred and the maximum of referred patients seen within two weeks, some patients need to wait to enter secondary care.  Across both models, in the PinPoint test arm, patients are prioritised to be seen in secondary within two weeks upon GP referral based on their risk of having cancer. With PinPoint test used as a prioritisation tool, high risk individuals are assigned a higher priority to be seen in secondary care within two weeks over medium risk and low risk individuals. In the standard care arm, there is no prioritisation rule therefore patients first referred first enter secondary care. In the context of PinPoint test as a triage and prioritisation tool, individuals who are categorised as low risk by the PinPoint test are initially not referred down to the urgent cancer pathway and only individuals classed as high and medium risk are referred to secondary care. Similar to the first use case, high risk individuals are seen first, followed by medium risk individuals  The diagnostic pathway in secondary care differs across urological models, specifically:   - **Prostate cancer model** – initial triage, testing with multiparametric magnetic resonance imaging (mpMRI) to identify patients at high risk of prostate cancer, and then transrectal ultrasound guided (TRUS) biopsy - **Bladder and kidney cancer model** – cystoscopy, ultrasound and x-ray.   All patients, regardless of arm, exit the models with or without a diagnosis of urological cancer. | |
| Base model logic | 2.2 | This section provides an overview of the logic common to both urological models – unless stated otherwise.  Patients suspected with urological cancer enter the models. A unique label identifier ‘Patient_ID’ is assigned to all new arrivals at the ‘Starting Point’ to track patients for internal validation purposes. For each new arrival, the following labels are assigned   - lbl_time at entry = to track when patients enter the model; - lbl_month entry = to track in what month patients have entered the model; - true_cancer_status = depending on the probability profile for disease prevalence, patients either (i) truly have urological cancer or (ii) truly do not have urological cancer.   In the bladder and kidney cancer model, each patient is assigned a label ‘lbl_cancer_referral’ reflecting if they were referred upon suspicion of bladder or kidney cancer based on a probability profile. This helped to assign each cancer patient who have to wait longer than 28 days to be seen in secondary care a decrement in 10-year survival specific to their cancer referral (e.g. prostate, bladder or kidney cancer), as part of the exploratory analysis.  Depending on the Simulation time, ‘Starting Point’ counts how many patients have entered the model in a given month. In the ‘Starting Point’, the simulation assigns an age group label to all patients positive to urological cancer based on a distribution/probability profile called ‘Age groups_cancer positive’. In the bladder and kidney cancer model, patients positive to either urological cancer are assigned an age group label conditional on the cancer they were initially referred to (e.g. patients truly with bladder cancer will be assigned a age group specific to bladder cancer). Across both models, the ‘Starting Point’ also assigns a label called ‘lbl_test’ depending on the intervention being evaluated to ensure that entities follow the correct diagnostic pathway in primary care.  In the ‘GP visit’ activity, the Routing Out On Work Complete performs the following tasks:   - A global variable counts the number of GP consultations; - In the bladder and kidney cancer model only, each patient is assigned a unique label called ‘lbl_time to request referral’ equal to Simulation time to track when the GP refers patients for further investigation in secondary care; - In the bladder and kidney cancer model only, the patient is routed out to either standard care or PinPoint test arm depending on the testing strategy being evaluated. - In the bladder and kidney cancer model only, in case standard care is being evaluated, this VL assigned each patient a minimum waiting time to be seen in secondary care equal to 13 days (13 days*480 minutes = 6240); - In the bladder and kidney cancer model only, global variables count the following model outputs: (i) All_Referrals; (ii) Cancer_Referrals; and (iii) No Cancer_Referrals.   In the prostate cancer model, patients visit the GP and undergo blood testing to check if their PSA levels are above the age-specific threshold and to get a PinPoint test result. In this model the ‘GP visit’ activity only counts the number of GP consultations. The ‘PSA testing’ performs the following tasks (see Routing Out On Work Complete):   - Each patient is assigned a unique label called ‘lbl_time to request referral’ equal to Simulation time to track when the GP refers patients for further investigation in secondary care; - The patient is routed out to either standard care or PinPoint test arm depending on the testing strategy being evaluated. - In case standard care is being evaluated, this VL assigned each patient a minimum waiting time to be seen in secondary care equal to one week (13 days*480 minutes = 6240); - Global variables count the following model outputs: (i) All_Referrals; (ii) Cancer_Referrals; and (iii) No Cancer_Referrals.   Focusing on the standard care arm, urgently referred patients enter ‘Minimum Waiting time’ activity and are assigned a minimum waiting time of 13 days across both urological models.  In the PinPoint test activity, the Routing Out On Work Complete performs the following tasks:   - A global variable counts the number of PinPoint tests run; - Each patient is assigned an individual risk of having cancer (‘high’, ‘medium’ or ‘low’ risk) depending on the individual’s true disease status and diagnostic sensitivity and specificity of the test. The PinPoint test result is recorded as an individual label and patients are routed out to either ‘High risk’, ‘Medium risk’ or ‘Low risk’ activity depending on the individual result. The logic first identifies high risk patients first using diagnostic accuracy for the rule-in use case [it applies sensitivity for patients truly with cancer and specificity for patients truly without cancer], and, out of those remaining, it distinguishes between medium and low risk using the diagnostic accuracy for the rule-out use case [it applies sensitivity for patients truly with cancer and specificity for patients truly without cancer]. Note that in this logic cumulative diagnostic accuracy estimates are used as each patient is assigned a single risk score with PinPoint test and compare it against a random number – therefore it is necessary to apply cumulative values to correctly distinguish high, medium and low risk patients. - While using PinPoint test as a prioritisation tool all patients are urgently referred. Each referred patient is assigned a minimum waiting time to be seen in secondary care equal to 13 days (13 days*480 minutes = 6240); - In the context of PinPoint test as a triage and prioritisation tool, individuals classed as low risk are initially not urgently referred whereas individuals with high and medium risk are urgently referred to secondary care for further investigation. All missed cases (i.e. patients truly with cancer who have been classed as low risk and therefore have not been initially urgently referred) are assumed (1) remain symptomatic; (2) visit the GP twice in the following six months; (3) experience a 6-month delayed referral to secondary care. Missed cases are then routed to’6 months-delayed referral(false-negative)’. Correctly identified low risk patients are routed out to an Exit Point called “Not Urgently Referred for cancer (true-negative) Exit Point”. The Exit Point performs the following tasks: - Count Costs by Disease Status’ VL; - ‘Store individual results’ VL - Global variables count the following model outputs: (i) All_Referrals; (ii) Cancer_Referrals; (iii) No Cancer_Referrals; (iv) C_Red Patients (sorted by true disease status); (v) C_Amber Patients (sorted by true disease status); (vi) C_Green Patients (sorted by true disease status); (vii) C_correctly prioritised PP; (viii) C_correctly referred PP; (ix) C_correctly NOT referred_PP; (x) C_correctly NOT prioritised_PP; (xi) C_incorrectly NOT referred_PP; (xii) C_incorrectly DE prioritised_PP.   In the context of PinPoint test used as triage and prioritisation tool, the ‘6 months-delayed referral (true-negative)’ activity performs the following tasks:   - Missed cases are assumed to experience a six-month delayed referral to be seen in secondary care. - Missed cases are assumed to experience an age-stratified six-month decrement to their 10-year survival. For each patient the logic checks the age group and then applies an age-stratified decrement in their 10-year survival. The logic then estimates the total life years lost by summing up the individual life years lost. - Count Costs by Disease Status’ VL; - ‘Store individual results’ VL - Missed cases then exit the model.   In the PinPoint test arm, patients are prioritised to be seen within two weeks in secondary care based on their risk score. The prioritisation takes place in ‘Queue for ‘Urgent referral for suspected cancer’”.  Across both testing arms, at the beginning of the 13^th^ waiting day, referred patients enter ‘Urgent referral for suspected cancer’ activity which assesses if a patient can be seen within two weeks depending on the maximum number of referred patients seen within two weeks. The Routing Out Work Complete of the ‘Urgent referral for suspected cancer’ activity performs the following tasks:   - Depending on the number of patients urgently referred and the maximum of referred urgently patients seen within two weeks, some patients need to wait to enter secondary care. Those patients seen within two weeks enter the queue “TWW target”.Where patients are not seen within two weeks, patients are assumed to experience an additional delay ranging between 1 additional day to a maximum of 16 days depending on the distribution of delayed days in which referred patients seen after two weeks – see ‘Additional Waiting Time’ VL (see section 2.4 for further detail. Patients who are not seen within two weeks wait the assigned additional waiting time (lbl_Minimum Waiting Time) in the queue ‘>14 days’ and, once the additional waiting time has expired, they enter secondary care clinic.   Referred patients then move to ‘Enter Secondary Care’ activity. In this activity, the Routing Out Work Complete performs the following tasks:   - Each patient is assigned an individual ‘lbl_time to enter clinic’ equal to Simulation time and the total time to referral is estimated as the (rounded) difference between time to enter the clinic minus the timepoint when the GP requested the referral; - Cancer patients seen after 28 days upon GP referral are assumed to experience a two-month delay decrement to their 10-year survival. In the ‘Calculation of delay-dependent life years lost’ VL, the logic first checks if a patient truly has cancer (true_cancer_status_ = 1) and then it checks if the time to referral (i.e. time difference between the timepoint when patient is seen in secondary care and the timepoint when the GP requested referral) exceeds 28 days. If so, for each patient the logic checks the age group and then applies an age-stratified decrement in their 10-year survival. The logic then estimates the total life years lost by summing up the individual life years lost.   In the ‘Enter Secondary Care’ activity, the label action VL performs the following tasks:   - Check if the patient met the two week waiting target (i.e. lbl_time to referral <= 6720 -1) [14 days * 480 minutes = 6720 minutes]; - Global variables count the number of patients seen within two weeks, sorted by (i) disease status; (ii) month of entry; and (iii) PinPoint test result in case PinPoint test is being evaluated; - If the patient has not met the two-week waiting target, the logic checks if the patient has been seen within 28 days (i.e. lbl_time to referral <= 13440 -1) [28 days * 480 minutes = 13440 minutes] or after 28 days; - Global variables count the number of patients seen after two weeks (i.e. ‘delayed referrals’), sorted by (i) disease status; (ii) month of entry; and (iii) PinPoint test result in case PinPoint test is being evaluated; - Global variables count the number of patients seen after 28 days sorted by disease status.   The following sections describe the diagnostic pathway in secondary care for each urological model, separately.  In the prostate cancer model, patients first move to the ‘Triage’ activity – where the Routing Out On Work Complete VL counts the number of patients undergoing triage. Patients then are tested with mpMRI. The Routing Out On Work Complete VL of the ‘mpMRI’ assigns a positive test result for patients truly with prostate cancer and a negative result for patients truly without prostate cancer as mpMRI is assumed to be 100% accurate. In addition to this, the activity performs the following tasks:   - Global variables count (i) number of mpMRI run; (ii) number of true positive result; (iii) number of true negative result; - Patients with negative mpMRI result are routed out to ‘No prostate cancer’ activity, whereas patients with positive mpMRI result are routed out to ‘Biopsy’ activity.   In the ‘No prostate cancer’ activity, the Routing Out On Work Complete VL entail the ‘Count Costs by Disease Status’ VL which estimates the costs in primary care, secondary care and total costs sorted by disease status. The Label VL runs the ‘Store individual results’ VL to paste the individual labels into a spreadsheet for internal validity checks.  In the ‘Biopsy’ activity, the Route in After Loading Work VL assigns a positive test result for patients truly with prostate cancer and a negative result for patients truly without prostate cancer as biopsy is assumed to be 100% accurate. The Routing Out On Work Complete VL performs the following tasks:   - Global variables count (i) number of biopsies run; (ii) number of multidisciplinary team cancer meetings; and (iii) number of unnecessary biopsies in case of patients truly without prostate cancer; - Count Costs by Disease Status’ VL   The Label VL runs the ‘Store individual results’ VL to paste the individual labels into a spreadsheet for internal validity checks.  In the bladder and kidney cancer model, both ‘Cystoscopy’ and ‘Ultrasound’ activities use global variables to count the number of cystoscopies, ultrasound and x-rays run, respectively, in the Routing Out On Work Complete VL.  In the ‘MDT’ activity, the Routing Out On Work Complete VL performs the following tasks:   - Global variable counts number of multidisciplinary team cancer meetings; - Count Costs by Disease Status’ VL; - ‘Store individual results’ VL   Across both models, the ‘End Run’ VL estimates the following outputs: (i) cost of GP consultations (sorted by disease status); (ii) additional testing cost primary care; (iii) cost of triage; (iv) cost of mpMRI; (v) cost of biopsies; (vi) cost of MDT; (vii) total cost in primary care (sorted by disease status); (viii) total cost in secondary care (sorted by disease status); and (ix) total costs (sorted by disease status). | |
| Scenario logic | 2.3 | We investigated the relative cost-effectiveness of PinPoint test across both use cases compared to usual care while capturing the impact of both (1) volume of referred patients to the urological diagnostic pathway; and (2) the efficiency of healthcare sites to see patients within two weeks upon GP referral. To do so, three scenarios were evaluated: high, low and average volume of patients referred to further diagnostic investigations in secondary care. For each volume scenario, we aimed to evaluate the impact of PinPoint test compared to standard care on highly efficient sites, average efficient sites and poorly efficient sites. This, in turn, resulted in a total of 9 scenarios (3 volume scenarios X 3 efficiency scenarios) explored for each testing strategy being evaluated.  We also explored how longer delays to diagnosis may impact upon health outcomes for those individuals with cancer. We applied estimates from a UK-based modelling study which assessed the impact of 2-, 4- and 6-month delays to referral on 10-year survival for different cancer subgroups (stratified by age) [6]. To apply these estimates, we used the age groups for each cancer pathway as reported in the Routes to Diagnosis data 2013-2016 [7] (**Table 1**). Life years lost were discounted at 3.5% per year based on the NICE discount rate.  In the models, individuals with cancer who have to wait longer than 28 days are assumed to experience a 2-month delay decrement to their 10-year survival due to the detrimental impact of waiting for cancer treatment. Similarly, in the triage and prioritisation use case, missed cases are assumed to experience a 6-month delay decrement to their 10-year survival following an incorrect low risk result. The total life years gained (LYG) were estimated comparing the total life years lost between standard care and PinPoint test arm.  In addition, to enable the standard cost-effectiveness calculations to be undertaken, we estimated the incremental net monetary benefit (INMB) comparing the PinPoint test against standard care, using the NICE willingness-to-pay (WTP) threshold of £20,000 per quality-adjusted life year (QALY) gained. There is currently, however, a lack of data on the impact of short-term delays to diagnosis of cancer on long-term health-related quality-of-life (HRQoL). We therefore assumed no quality-of-life impact of delayed or faster diagnosis for patients with cancer – the QALYs gained are therefore based solely on the LYG. | |
| Algorithms | 2.4 | See Technical documentation - available upon request  This section explains the following simulation logics:   1. Estimation of patients entering the model each model & clock properties   In both models, on the first minute (9:00am) of the first day of each month, a certain cohort of patients enter the ‘Start Point’ depending on the month-specific number of patients referred to the suspected urological cancer pathway. The timing of ‘Start Point’ is set equal to a global variable called ‘X1’ which enables the new patient arrivals only once a month. The global variable X1 reflects the SIMUL8 minutes required until the beginning of the next month depending on the number of working days in the month being simulated. Based on the NHS England Waiting Time Statistics, it is unknown the exact day when patients are urgently referred to secondary care for further cancer investigation. It is therefore assumed that at the beginning of each month a cohort of patients with a different size enters the models  Using the ‘Time Check Logic’ VL every minute, the simulation identifies what month is being simulated based on the Simulation time global variable and the total (cumulative) number of working days in each month. Once the current month is identified, the ‘Inter arrival time and maximum daily capacity calculation’ VL defines the number of patients entering the models in that given month and maximum number of patients seen within two weeks depending on (i) the volume of referrals and (ii) level of efficiency being simulated. The VL then reschedules the next arrival through the ‘Start Point’ based on the duration of the month of interest (X1). This, in turn, allows for the models to have new patient arrivals at the beginning of every month. In addition to this, at the beginning of each month, the global variable ‘Patient Entering Clinic’ is reset to 0 to properly simulate the ability of the secondary care clinic to see patients within two weeks upon GP referral based on the monthly NHS England Waiting Time data.   1. ‘Additional Waiting Time’ VL   This logic applies to patients who are not seen in secondary care within two weeks. The simulation checks in what month the individual patient has entered the model. Based on the month of entry, volume of referrals and level of efficiency, for each patient the simulation sets the additional waiting time category (i.e. lbl_waiting time group) equal to the categorical month-specific distribution of additional delay, including (i) 15 to 16 days (which correspond to 1 to 2 additional waiting days); (ii) 17 to 21 days (which correspond to 3 to 7 additional waiting days); (iii) 22 to 28 days (which correspond to 8 to 14 additional waiting days); and (iv) after 28 days (which correspond to 15 to 16 additional waiting days). Note that a maximum of 30 days to be seen in secondary care assumed since the NHS England Waiting Time Statistics only reports the number of patients seen after 28 days without specifying the upper bound.  Because the NHS England Waiting Time Statistics reported additional waiting days categorically only, a uniform distribution was applied to estimate the additional waiting days for patients so that each value within the range was assumed to be likely to occur. For each range, the simulated additional waiting time was   - 15 to 16 days (which correspond to 1 to 2 additional waiting days) = U(480, 959) - 17 to 21 days (which correspond to 3 to 7 additional waiting days) = U(960, 3359) - 22 to 28 days (which correspond to 8 to 14 additional waiting days) = U(3360, 6719) - after 28 days (which correspond to 15 to 16 additional waiting days) = U(6720, 7679) | |
| Components | 2.5 | 2.5.1. Entities | Both models track adult patients visiting their GP with symptoms indicative of urological cancer who meet the National Institute for Health Care Excellence (NICE) NG12 two week referral criteria for further investigation in secondary care – either high prostate antigen specific (PSA) or haematuria for prostate cancer and kidney and bladder cancer, respectively. |
|  |  | 2.5.2. Activities | The key activities for both models are presented in Figure 1 and described in section 2.1 and 2.1.  Each activity is replicated for many times (i.e. 1,000) to ensure that there are no bottlenecks in the urological models. |
|  |  | 2.5.3. Resources | No resources were applied to the urological models. |
|  |  | 2.5.4. Queues | All queues in the models follow a first in first out discipline, except the queue called “Queue for ‘Urgent referral for suspected cancer”. This queue only applies to the PinPoint test arm where patients are prioritised based on their risk of having cancer. In this queue, patients are prioritised based on the ‘PP_risk’ label indicating their risk of having cancer – 3 indicating high risk (‘red-flagged’), 2 indicating medium risk (‘amber-flagged’) and 1 indicating low risk (‘green-flagged). |
|  |  | 2.5.5. Entry/Exit points | Entry and Exit points for both models are presented in **Figure 1**.  In both urological models, on the first minute (9:00 am of the first day of each month) a certain cohort of patients enters the model at the ‘Start Point’ (illustrated as a blue arrow to the left of the ‘GP activity’ in **Figure 1**). The size of the cohort each month depends on the month-specific number of patients referred to the suspected urological cancer pathway. For more detail see section 2.4.  In both models, in the context of PinPoint test as triage and prioritisation tool, individuals classed as low risk exit the model at either ‘Not Urgently Referred for cancer (true-negative) Exit Point’ or ‘Missed Case Exit’ depending on the true disease prevalence.  In the prostate cancer model (across both use cases), all patients exit the model at ‘Prostate Cancer Detected Exit’ or ‘No Prostate Cancer Detected Exit’ depending on the final diagnosis of prostate cancer. In the bladder and kidney cancer model (across both use cases), all patients exit the model at ‘Exit point’ regardless of the final diagnosis. |
| 1. **Data** |  | | |
| Data sources | 3.1 | A list of input parameters and associated data sources can be found in Table 1 and Supplemental Material A.  To populate both models, we used the main data sources, including:   - NHS England Two Week Waiting Time Statistics – to inform the number of referred patients, the number of referred patients seen within two weeks and the number of patients seen after two weeks - Route to Diagnosis dataset was used to inform the prevalence of urological cancer, and the proportion of urological referrals specific to prostate cancer, bladder and kidney cancer, separately. - NHS Reference Costs, NHS National tariffs 2020/2021 and Personal Social Services Research Unit (PSSRU) – to inform unit costs | |
| Pre-processing | 3.2 | Analysis was undertaken on (i) the number of patients referred to the suspected urological cancer pathway; (ii) the number of referred patients seen within two weeks; (iii) the number of patients seen in 15 to 16 days; (iv) the number of patients seen in 17 to 21 days; (v) the number of patients seen in 22 to 28 days; and (vi) the number of patients seen after 28 days based on the NHS England Waiting Time Statistics.  Healthcare providers were categorised both by volume of referrals (high, average, and low) and by efficiency. After ranking the providers by number of referrals for each month, those in the top 40 were categorised as ‘High volume’ providers, those in the bottom 40 as ‘low volume’ providers and the remaining were categorised as ‘average volume’. Within each volume subgroup (i.e. ‘high volume’, ‘average volume’, ‘low volume’), for each month we then identified the top and bottom 25% performing healthcare sites based on the reported percentage of referred patients seen within two weeks as stated in the NHS Waiting Time Statistics. For each volume and efficiency subgroup, we then calculated the following outputs: (i) the average number of referred patients seen by a specialist; (ii) the average number of referred patients seen within two weeks; and (iii) the average number and proportion of referred patients seen after two weeks (15 to 16 days; 17 to 21 days; 22 to 28 days; after 28 days). | |
| Input parameters | 3.3 | A list of input parameters for both models is provided in Table 1. | |
| Assumptions | 3.4 | Model assumptions common to both models are listed below, including:   1. GPs are assumed to fully adhere to the results of the PinPoint test; 2. Upon referral for further investigations in secondary care, patients have to wait a minimum of 13 before they are booked for further investigations in secondary care; This is due to (i) logistical reasons (e.g. booking, phone calls) and (ii) based on the NHS England Waiting Time Statistics, it is unknown exactly the day within two weeks patients are actually seen in secondary care; 3. Each month, a limited number of patients can be seen in secondary care within 2 weeks. Once the clinic reaches the maximum, individuals who did not get an appointment will wait additional days (between one to 16 days), depending on the ability of the providers to meet the TWW target. 4. Patients with urological cancer (either true-positive or false-negative) who have waited longer than 28 days to be seen in secondary care are exposed to a reduced 10-year survival due to the detrimental impact of waiting for cancer treatment and/or surgery for two months. The longer the delay patients experience, the greater the 10-year survival for patients with urological cancer. 5. In the triage and prioritisation use case, cancer patients incorrectly not referred are assumed to remain symptomatic, visit the GP twice for additional consultations and receive a delayed referral after 6 months. As a result, they are assumed to experience an age-stratified six-month delay decrement to their 10-year survival due to the initial correct diagnosis and the extended waiting time. 6. In the triage and prioritisation use case, no benefits or harms associated with receiving a true-negative result with PinPoint test (i.e. individuals without cancer classified as low risk) were assumed due to (i) paucity of data on the expected health impact and management of individuals initially not urgently referred using PinPoint as a triage and prioritisation tool; (ii) the models focus on the processes and expected impact of PinPoint test of patients referred down the urgent suspected cancer pathway only. 7. All patients with positive test results for cancer in secondary care undergo a multidisciplinary cancer meeting to confirm cancer diagnosis.   There are also some clinical assumptions specific to the prostate cancer model, including  1. All tests run in secondary care required are assumed available if needed when a patient is seen in secondary care.  2. All tests run in secondary care are assumed to have perfected diagnostic accuracy in identifying patients with urological cancer and ruling out patients without cancer  3. An MDT meeting confirms the cancer diagnosis for all patients referred to secondary care.  4. The models do not include the treatment pathways (and associated costs) as PinPoint test is not expected to impact upon treatment decisions, just the timing of diagnosis and treatment. | |
| 1. **Experimentation** |  | | |
| Initialisation | 4.1 | No warm up period was applied as the model applies monthly-specific data for the number of patients entering the simulation. The model runs in minutes. | |
| Run length | 4.2 | The models cover a one-year period between 1^st^ December 2021 and 30^th^ November 2022 (295 days to ensure that all patients exit the simulation). This time horizon was selected to explore the impact that PinPoint test would have had on reducing waiting time for referred patients after the social distancing measures used during the COVID-19 pandemic were lifted. | |
| Estimation approach | 4.3 | All model analyses were based on running 650 replications (trials) of the model, to account for first order uncertainty – each run using a different random number sequence. | |
| 1. **Implementation** |  | | |
| Software or programming language | 5.1 | The models were developed in Simul8 (SIMUL8 Corp, Boston, MA)([https://www.simul8.com](https://www.simul8.comD)). | |
| Random sampling | 5.2 | All model analyses were based on running 650 replications of the model, to account for first order uncertainty – each run using a different random number sequence. | |
| Model execution | 5.3 |  | |
| System Specification | 5.4 | The models were implemented in the commercial software SIMUL8 version 28.0.0.4249 Student edition, and run on a VivoBook ASUS laptop X571GT_F571GT with a 2.60 GDHz Intel ® Core ™ i7 processor and 16 GB of memory under Microsoft Windows 10 Home (build 19,043). Total model run time was approximately 23 minutes for both testing strategies without using parallel processing. The model runs from 9am to 5pm (8 hours = 480 minutes in SIMUL8) for 5 days each week (Monday to Friday). | |
| 1. **Code Access** |  | | |
| Computer Model Sharing Statement | 6.1 | Simul8 software can be purchased via the Simul8 website: [https://www.simul8.com](https://www.simul8.comD). Model coding can be requested to the corresponding author at [P.Cocco@leeds.ac.uk](mailto:P.Cocco@leeds.ac.uk) | |

1. **Husereau D, et al.** Consolidated Health Economic Evaluation Reporting Standards 2022 (CHEERS 2022) Statement: Updated Reporting Guidance for Health Economic Evaluations. *Value in Health*. **2022**

2. **Monks T, et al.** Strengthening the reporting of empirical simulation studies: Introducing the STRESS guidelines. *Journal of Simulation*. **2019**

3. **Brennan A, et al.** A taxonomy of model structures for economic evaluation of health technologies. *Health Econ* **2006**

4. **Karnon J, et al.** Selecting a decision model for economic evaluation: a case study and review. *Health Care Manag Sci*. **1998**

5. **Squires H, et al.** A Framework for Developing the Structure of Public Health Economic Models. *Value in Health*. **2016**

6. **Sud A, et al.** Effect of delays in the 2-week-wait cancer referral pathway during the COVID-19 pandemic on cancer survival in the UK: a modelling study. *The Lancet Oncology*. **2020**

7. **National Cancer Registration and Analysis Service (NCRAS), et al.** Routes to diagnosis 2013-2016 2018. Available online: <http://www.ncin.org.uk/publications/routes_to_diagnosis>. (accessed 16/11/2022)
