# Supplementary material for: The National Health Service urgent cancer referral pathway for suspected urological cancers: early economic evaluation of a risk prediction test
Source: Int J Technol Assess Health Care. 2024 Jan 12;40(1):e9. doi: 10.1017/S0266462324000023 (PMC10859831; doi:10.1017/S0266462324000023)
Supplement: Cocco et al. supplementary material 3 — Cocco et al. supplementary material [file S0266462324000023sup003.docx]

# Supplementary File 3 – supplementary results

## Prostate cancer model

### Deterministic results

**Supplementary Table 3. 1 Results for prostate cancer model: average number of patients entering the model, average estimates for referral patterns (%) for standard care and PinPoint test (both use cases) across 650 deterministic model replications, in the context of providers with high and average volume of referrals, sorted by performance scenarios**

**Supplementary Table 3. 2** **Results for prostate cancer model: average number of patients entering the model, average estimates for referral patterns (%) for standard care and PinPoint test (both use cases) across 650 deterministic model replications, in the context of providers with low volume of referrals, sorted by performance scenarios**

**Supplementary Table 3. 3 Full deterministic results for prostate cancer model for each testing strategy in the context high volume referrals sites, sorted by efficiency levels**

**Supplementary Table 3. 4 Full deterministic results for prostate cancer model for each testing strategy in the context average volume referrals sites, sorted by efficiency levels**

**Supplementary Table 3. 5 Full deterministic results for prostate cancer model for each testing strategy in the context low volume referrals sites, sorted by efficiency levels**

### Probabilistic results – 1200 PSA runs

## Bladder and kidney cancer model

### Deterministic results

**Supplementary Table 3. 6** **Results for bladder and kidney cancer model: average number of patients entering the model, average estimates for referral patterns (%) for standard care and PinPoint test (both use cases) across 650 deterministic model replications, in the context of providers with high and average volume of referrals, sorted by performance scenarios**

**Supplementary Table 3. 7** **Results for bladder and kidney model: average number of patients entering the model, average estimates for referral patterns (%) for standard care and PinPoint test (both use cases) across 650 deterministic model replications, in the context of providers with low volume of referrals, sorted by performance scenarios**

**Supplementary Table 3. 8 Full deterministic results for bladder and kidney cancer model for each testing strategy in the context of high volume referrals sites, sorted by efficiency levels**

**Supplementary Table 3. 9 Full deterministic results for bladder and kidney cancer model for each testing strategy in the context of average volume referrals sites, sorted by efficiency levels**

**Supplementary Table 3. 10 Full deterministic results for bladder and kidney cancer model for each testing strategy in the context of average volume referrals sites, sorted by efficiency levels**

### Probabilistic results – 1200 PSA runs
